# Dosimetric feasibility of lattice radiotherapy for bulky tumors: a paired VMAT vs. helical tomotherapy comparison - the STRYKE trial

**DOI:** 10.3389/fonc.2026.1986573

**Published:** 2026-10-27

**Authors:** Mottareale R, Serra M, Pezzulla D, Cilla S, Borzillo V, Scipilliti E, Silvestro G, De Palma G, Ametrano G, Buonanno F, Arrichiello C, d’Alesio V, di Franco MA, Mercogliano S, Cuomo A, Cavalcanti E, Maranzano E, Donati C, Cellini F, Ravo V, Di Franco R

**Affiliations:** 1 Department of Radiation Oncology, Istituto Nazionale Tumori - IRCCS Fondazione “G. Pascale”, Napoli, Italy; 2 Radiation Oncology Unit, Responsible Research Hospital, Campobasso, Italy; 3 Medical Physics Unit, Responsible Research Hospital, Campobasso, Italy; 4 Division of Anesthesia and Pain Medicine, Istituto Nazionale Tumori - IRCCS Fondazione “G. Pascale”, Napoli, Italy; 5 Laboratory Medicine Unit, Istituto Nazionale Tumori - IRCCS Fondazione “G. Pascale”, Napoli, Italy; 6 University of Perugia, Perugia, Italy; 7 Radiation Oncology, IRCCS Azienda Ospedaliero-Universitaria di Bologna, Bologna, Italy; 8 Department of Medical and Surgical Sciences (DIMEC), Alma Mater Studiorum University of Bologna, Bologna, Italy; 9 Dipartimento di Diagnostica per Immagini, Fondazione Policlinico Universitario “A. Gemelli” IRCCS, UOC di Radioterapia Oncologica ed Ematologia, Rome, Italy

**Keywords:** bulky tumors, helical tomotherapy, lattice radiotherapy, SFRT, VPDR

## Abstract

**Background:**

Lattice Radiotherapy (LRT) delivers ablative doses to intratumoral vertices alternating with low-dose valleys to treat bulky tumors. While VMAT-LRT is widely adopted, Helical Tomotherapy (HT-LRT) may offer enhanced spatial dose modulation. This study provides the first paired dosimetric comparison of HT-LRT versus VMAT-LRT across scalar, directional, and DVH-based endpoints.

**Methods:**

Paired HT-LRT and VMAT-LRT plans were evaluated in a cohort of 22 patients with bulky tumors using a staggered triangular lattice geometry (5.0 cm inter-vertex spacing, 1.0 cm diameter). Spatial dose modulation was characterized using three scalar Valley-to-Peak Dose Ratio (VPDR) computational methods (VPDR_Wu_, VPDR_D90/D10_, VPDR_median_) and a directionally resolved decomposition along principal and diagonal spatial axes. Target coverage, OAR sparing, and morphological correlations were systematically analyzed.

**Results:**

HT-LRT demonstrated statistically significant superiority over VMAT-LRT across all scalar VPDR metrics (*p* < 0.05). Directional analysis revealed that HT-LRT advantage is anisotropic, concentrating along intra-plane radial and principal axes, whereas oblique inter-plane diagonals showed comparable modulation between techniques. HT-LRT yielded significantly cooler valley doses and improved low-dose OAR sparing without compromising target coverage (*D*_95%_). Target elongation positively correlated with radial VPDR in both modalities due to inter-plane dose summation, while compactness influenced radial VPDR exclusively in HT-LRT.

**Conclusion:**

HT-LRT should provide superior, directionally selective spatial dose modulation compared with VMAT-LRT in bulky tumors. Directionally resolved VPDR analysis exposes technique-specific geometric strengths obscured by scalar summary metrics, supporting HT-LRT as a promising platform for LRT execution, while the relative contribution of delivery technique and planning strategy warrants further investigation.

## Introduction

Bulky and locally advanced tumors, frequently unsuitable for surgical resection or systemic treatment alone, are commonly managed with radiotherapy as a minimally invasive option capable of achieving durable local control while preserving quality of life (1). Contemporary external-beam platforms, most notably Volumetric Modulated Arc Therapy (VMAT) and Helical Tomotherapy (HT), have substantially improved the achievable balance between target coverage and normal-tissue sparing, attributable, respectively, to continuous intensity modulation during gantry rotation and to a helical, CT-like delivery geometry well suited to large and irregularly shaped volumes (1–3). However, in bulky or anatomically constrained disease, this conformal paradigm meets intrinsic limitations: the tolerance of adjacent organs at risk frequently precludes uniform dose escalation across the entire target, forcing a compromise between tumoricidal coverage and acceptable toxicity and leaving biologically resistant subregions effectively under-treated even when nominal coverage metrics appear satisfactory (4–6). Furthermore, irradiating large tumor masses remains challenging due to the presence of hypoxic, radioresistant subvolumes that require radiation doses beyond safe normal-tissue tolerances (7). Consequently, viable tumor cells may survive even well-planned, homogeneous treatments. Additionally, delivering high physical doses across extensive tissue areas can disrupt local vascular and stromal architecture (8). This vascular damage exacerbates tumor hypoxia, impairs the delivery of systemic therapeutic agents, and substantially dampens the host anti-tumor immune response by hindering the trafficking of tumor antigens, lymphocytes, and dendritic cells (6). Notably, stereotactic approaches such as SRS and SBRT have demonstrated that durable tumor control does not require strict physical dose homogeneity. These techniques are typically delivered with intentionally non-uniform dose distributions, featuring central high-dose cores with steep peripheral fall-off, while achieving excellent local control, in part through biological mechanisms extending beyond direct cytotoxicity (9, 10). Against this backdrop, Spatially Fractionated Radiation Therapy (SFRT) emerges as a compelling alternative (11). Originally conceived to circumvent skin-dose limitations associated with orthovoltage equipment, SFRT has been subsequently refined with modern linear accelerators into a technique capable of safely delivering highly localized ablative dose peaks, a strategy particularly valuable in palliative scenarios requiring rapid symptom control (12–14). Unlike conventional planning, which seeks uniform dose distribution throughout the target, SFRT deliberately sculpts a non-uniform intratumoral dose landscape, alternating focal high-dose regions, commonly known as vertices or peaks, with low-dose zones, or valleys, separated by steep spatial dose gradients (11–13). Its rationale, however, extends beyond direct cytotoxicity within high-dose subvolumes. Proposed mechanisms include bystander signaling in non-targeted cells (14), disruption of the intratumoral vasculature within peak regions (6, 11), and, of particular relevance given the current prominence of immunotherapy, induction of a systemic anti-tumor immune response (15), wherein the irradiated lesion acts as an *in situ* immunogenic vaccine capable of inducing systemic regression in non-irradiated distant lesions (the abscopal effect) (18–20). Within the SFRT paradigm, its three-dimensional implementation, known as Lattice Radiotherapy (LRT), was first introduced by Wu and colleagues in 2010 (16). LRT relies on advanced intensity-modulated delivery to generate a volumetric arrangement of discrete intratumoral hot spots, defining the vertex target volume (VTV) (12). Promising clinical experiences have been reported in retrospective series and case reports across various disease sites, including soft-tissue sarcoma (17), non-small cell lung cancer (18), and head-and-neck malignancies (19). The observed favorable symptomatic responses and measurable tumor volume reductions position LRT as a viable therapeutic recourse when conventional dose-homogeneous approaches are either infeasible or inadequate. A framework consolidating the physical and dosimetric principles of LRT, together with practical planning guidance, was subsequently formalized by Wu et al. in 2020 (12); This study identifies stereotactic-capable delivery systems such as arc-based linear accelerators equipped with flattening filter-free (FFF) beams, or dedicated robotic platforms such as CyberKnife, as the technologies most compatible with the three dimensional implementation of Lattice SFRT (12). Notwithstanding these promising results, the widespread clinical implementation of SFRT continues to face significant challenges, including the requirement for high precision in treatment planning and dosimetry verification, the absence of standardized treatment protocols across institutions, and an incomplete understanding of the underlying biological mechanisms. A recent systematic review from our group (20), confirmed that VMAT-based LRT currently represents the predominant approach reported in the literature, owing to the widespread availability of FFF linear accelerators and the mature optimization frameworks developed for this delivery modality. In contrast, only two published studies to date have investigated the potential of Helical Tomotherapy for LRT implementation, both conducted in phantom-based dosimetric settings (21, 22). Despite these promising phantom-based dosimetric results, no structured dosimetric comparison between VMAT FFF and Helical Tomotherapy for patient-specific LRT planning has been reported in the literature.

The primary aim of the present study is to address this gap through a retrospective dosimetric comparison between the two delivery modalities across a cohort of patients with bulky tumors at heterogeneous anatomical sites, in order to characterize the effective dosimetric performance of Helical Tomotherapy for LRT relative to the established VMAT FFF approach.

A secondary aim of this study concerns the methodological characterization of the VPDR. The marked heterogeneity in VPDR values reported across published LRT experiences is largely attributable to the diversity of computational approaches employed, rather than to genuine differences in dosimetric performance between institutions (20). To address this, three distinct VPDR extraction methodologies were systematically applied and compared on the same patient cohort: the pairwise midpoint approach proposed by Prado et al. (23), the DVH-based D90%/D10% ratio described by Borzov et al. (24), and the mean low-dose percentile approach defined by Wu et al. (12, 25). Applying all three methods to identical planning data allows their mutual consistency to be assessed and provides a methodologically transparent basis for cross-institutional comparisons.

Beyond the choice of computational method, a further source of variability in reported VPDR values resides in the directional sampling of dose profiles. In any three-dimensional staggered lattice geometry, inter-vertex distances are structurally anisotropic: vertex pairs lying within the same transverse plane and those connecting adjacent planes subtend geometrically distinct distances, with systematically different valley dose levels as a consequence. Rather than collapsing this directional variability into a single scalar average, which would obscure physically meaningful differences in peak-valley dose modulation, the present study proposes a systematic directional decomposition of the VPDR. Linear dose profiles were extracted along the principal anatomical axes, radial directions, and coronal and sagittal diagonal trajectories, yielding a directionally-resolved characterization of the dose modulation pattern for each delivery technique. This framework does not eliminate an intrinsic geometric property of three-dimensional lattices, but renders it explicitly quantifiable, enabling a more rigorous and reproducible assessment of LRT dosimetric quality across institutions and delivery platforms.

## Materials and methods

### Patients and clinical characteristics

This retrospective study evaluated a cohort of 22 consecutive adult patients (*n* = 22) with bulky tumor lesions originally treated with external beam radiotherapy (EBRT) at our Institution between April 2019 and December 2025. All cases were retrospectively replanned using Lattice Radiotherapy (LRT) as part of the STRYKE Trial (Protocol No. 83/25 OSS).

The cohort represents a heterogenous clinical spectrum encompassing soft tissue sarcomas of the extremities and abdomen/pelvis, mediastinal/thoracic carcinomas, gynecological malignancies, and melanoma. Over half of the cases presented with local tissue infiltration (54.5%), and the majority harbored high-grade histology (63.6%).

Treatment intent was categorized into neoadjuvant (n = 11, 50.0%) and non-neoadjuvant/palliative regimens (n = 11, 50.0%). To better reflect clinical practice, palliative indications were further sub-classified into general palliative/symptomatic control (n = 9, 40.9%), cytoreductive-only intent (n = 1, 4.5%), and acute hemostatic treatment (n = 1, 4.5%). Patients in the palliative and hemostatic cohorts represent a high-vulnerability population characterized by advanced disease stages and significantly compromised overall life expectancy. In this real-world setting of limited survival prognosis, non-neoadjuvant intent strongly dictated the choice of treatment schedule, characterized by a substantial de-escalation of both total physical dose and overall number of fractions. While neoadjuvant cases uniformly received standard normofractionated schedules (50 Gy in 25 fractions, median $BED_{10}=60.0\text{ Gy}$), palliative and hemostatic courses utilized short-course, hypofractionated or single-fraction regimens ranging from 20–30 Gy in 4–10 fractions (median $BED_{10}=30.0\text{ Gy}$) down to single-fraction regimens (8 Gy in 1 fraction for acute bleeding control, $BED_{10}=14.4\text{ Gy}$).

### Simulation and contouring

Computed tomography simulation was performed using a Canon Aquilion scanner (voxel spacing: 3.0 × 1.367 × 1.367 mm). Structure delineation was performed in MIM (GE HealthCare), with organs at risk contoured using the integrated auto-contouring module to ensure consistency across cases. Gross tumor volume (GTV) delineation was independently performed/reviewed by two experienced radiation oncologists (R.D.F. and G.D.P.).

### Prescription and planning objectives

To ensure comparability across the retrospective dosimetric analysis, a uniform prescription dose of 21 Gy in three fractions was applied to all LRT plans. The prescription was chosen as a compromise between our institutional clinical experience with SBRT and published three-fraction LRT experiences ranging vertex doses between 21–27 Gy (20). For both techniques, planning was performed with the explicit objective of generating the steepest and most spatially uniform dose gradient within the PTV-Lattice, an aspect that is frequently overlooked in published LRT experiences, where priority is given to maximizing peak dose without constraining valley dose homogeneity, to assure the appropriate directional dependence of the VPDR evaluation. For both techniques, the maximum dose within the VTV was allowed up to 120% of the prescription dose, in keeping with the intentionally heterogeneous, ablative peak dose distribution of LRT. All VMAT-LRT and HT-LRT plans were optimized by the same planner using the standardized templates described below, and each plan was independently reviewed by two experienced medical physicists.

### Helical tomotherapy planning

HT-LRT plans were generated using the Accuray Precision^®^ treatment planning system (version 3.3.1.3) with a Convolution-Superposition dose calculation algorithm and delivered on an Accuray Radixact^®^ system. Plans were configured in fixed jaw mode with a field width of 2.5 cm and a pitch of 0.187. The dose grid resolution was 2.73 × 3.00 × 2.73 mm. Delivery mode was helical IMRT without motion tracking. Leaf open/close time cut-off was set to 20 ms. Median gantry period was 27.30 s (range 16.60–42.30 s), median modulation factor was 2.25 (range 1.70–2.80), and median number of active rotations was 35.85 (range 22.90–66.60). All plans were generated in fixed-jaw mode, the only jaw configuration available on our system; dynamic jaw modulation, which may further improve dose conformality and reduce treatment time, was not evaluated in this study and represents a promising direction for future investigation. A standardized planning template was applied across all cases to ensure consistency and promote steep dose gradients, with the following objectives: VTV minimum dose 21 Gy; PTV-Lattice maximum dose< 11 Gy; normal tissue objective (NTO) fall-off function applied to the External structure with the following constraints: 100% of the prescription dose at 5 mm, 30% at 10 mm, and 10% at 20 mm from the target surface.

### VMAT FFF planning

VMAT-LRT plans were generated in RayStation^®^ using a Collapsed Cone dose calculation algorithm with a 3 × 3 × 3 mm dose grid, and delivered on a VersaHD linear accelerator (Elekta AB, Sweden) equipped with an Agility MLC (Elekta AB, Sweden). Each plan employed a four-arc configuration distributed across two isocentres, with collimator angles ranging from 10° to 350°. A ring-based planning template was applied uniformly across cases, with the following dose objectives: VTV minimum dose 21 Gy; PTV-Lattice maximum dose< 11 Gy; Ring 1 (PTV + 1 cm expansion) dose fall-off from 9 Gy to 7 Gy over 1 cm; Ring 2 (Ring 1 + 1 cm expansion) dose fall-off from 7 Gy to 5 Gy over 1 cm; External structure (body contour excluding PTV and Ring 2) maximum dose< 5 Gy.

### Lattice delineation and dose profile extraction

Lattice delineation was performed semi-automatically using a dedicated in-house Python-based (version 3.12) workflow implemented with the SimpleITK and RTStructBuilder libraries. Starting from GTV contour exported in DICOM RT-STRUCT format, an isotropic morphological erosion of 2 cm was applied to generate a reduced inner volume, referred to as the lattice volume, within which vertex placement (VTV) was constrained, thereby ensuring a minimum geometric margin between vertex boundaries and the outer target surface. Vertex positions were distributed according to a staggered three-dimensional triangular grid, defined by a uniform center-to-center spacing (D_c-c_) of 5 cm, considered both on the in-plane and inter-plane directions (**Figure 1**). Each vertex of the VTV was modelled as a sphere of 5 mm radius (1.0 cm diameter), and only spheres whose center fell within the eroded lattice volume were retained. The resulting vertex configuration was exported as a new RT-STRUCT file and transferred to the TPS for dose optimization (**Figure 2**). The lattice array design adopted in this study reflects one of the most commonly reported configurations in the current literature, in a field where no standardized approach has yet been established (23, 26, 27).

**Figure 1:**
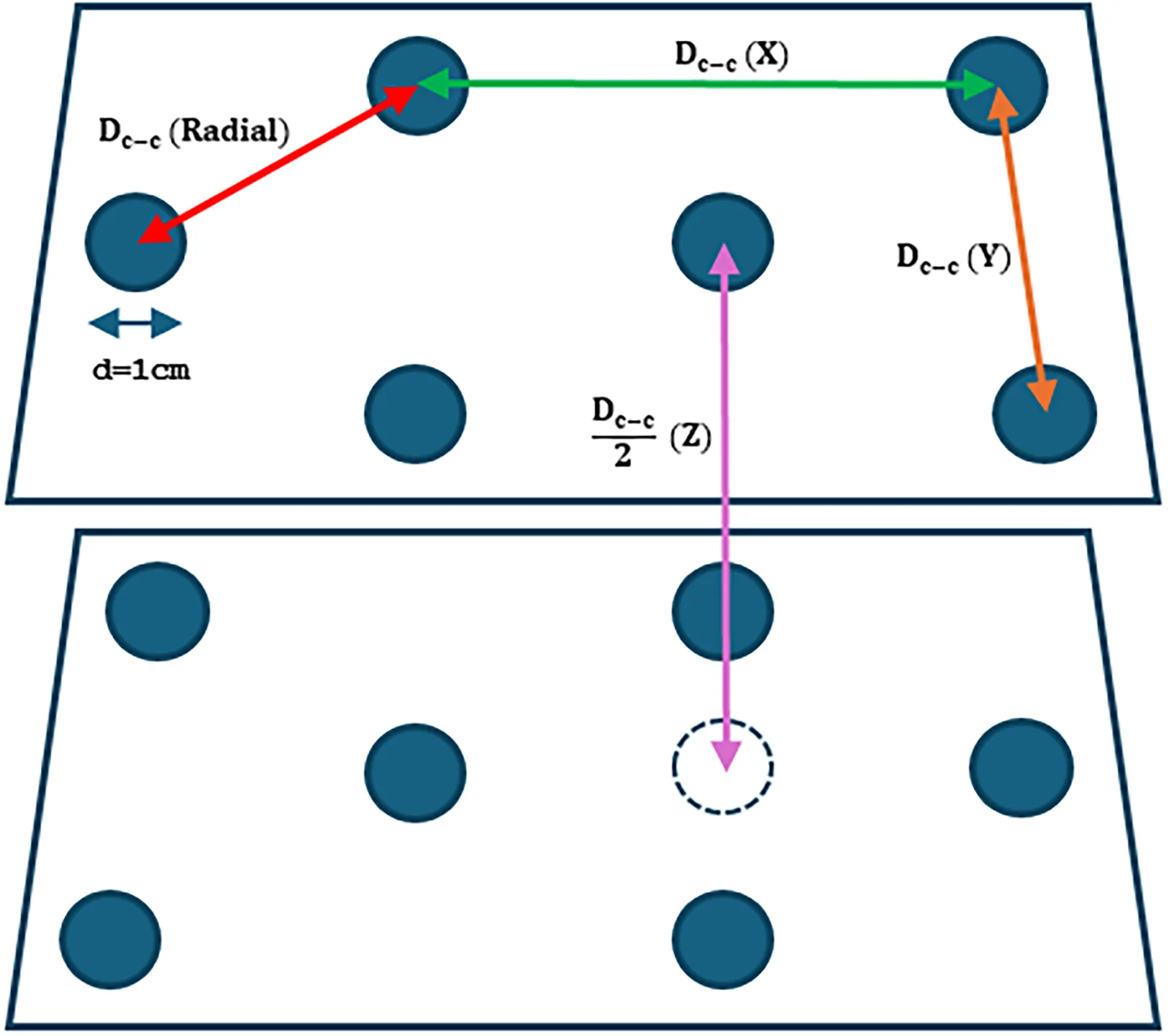
Staggered triangular lattice geometry and directional classification of center-to-center vertex distances (Dc-c). Upper and lower panels: two adjacent transverse planes; filled circles: vertices (d = 1 cm). Arrows: radial (red), X, left–right (green), Y, anterior–posterior (orange) Dc-c; inter-plane Z, superior–inferior (magenta) corresponding to (½) Dc-c; dashed circle: projection of an upper-plane vertex onto the lower plane. Coronal and sagittal diagonal distances are shown in **Figure 2**.

**Figure 2:**
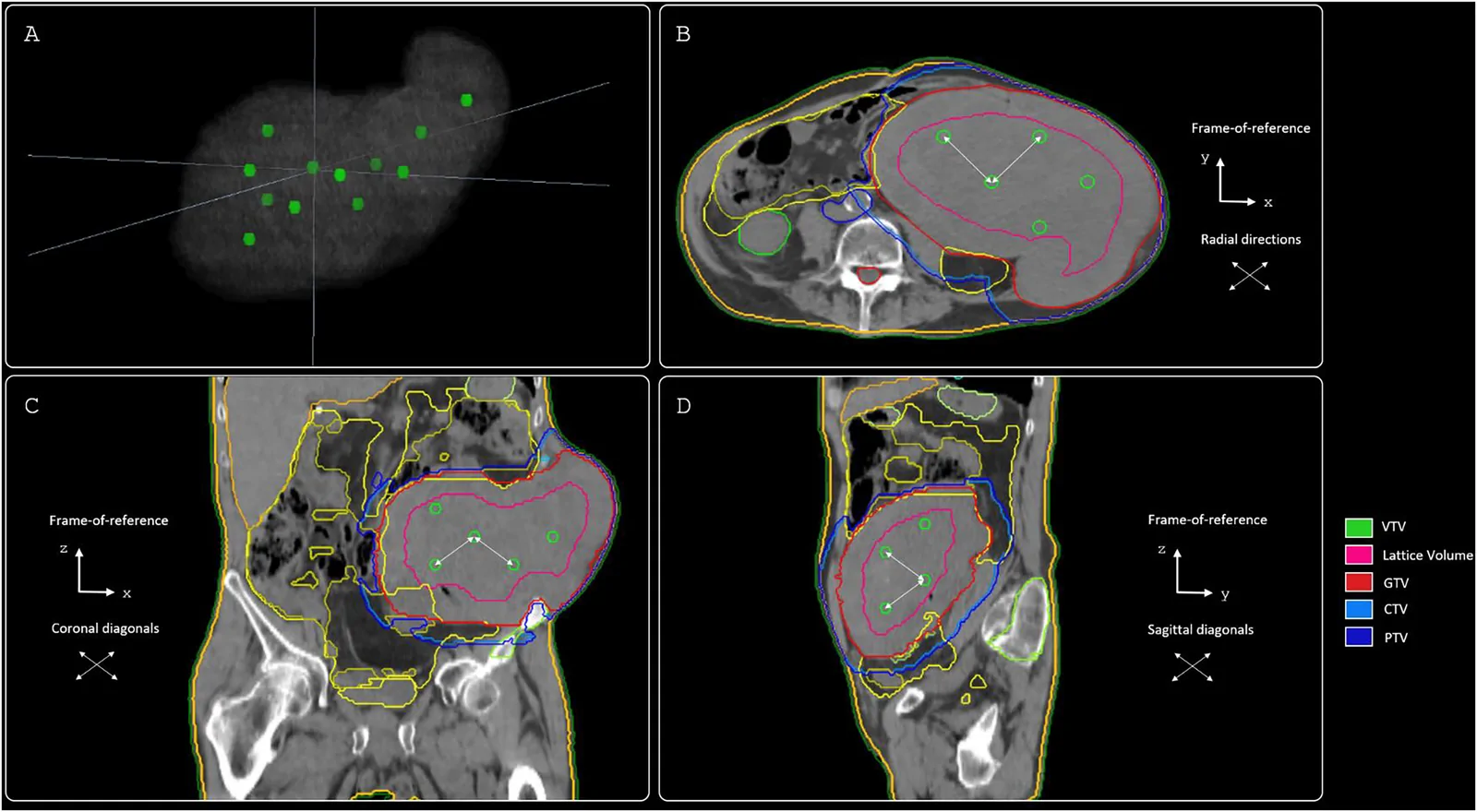
Directional classification framework for center-to-center distance assessment in a clinical lattice radiotherapy representative case. **(A)** Three-dimensional rendering of the VTV within the GTV, displayed with the anatomical frame of reference. **(B)** Axial (x–y), **(C)** coronal (x–z) and **(D)** sagittal (y–z) CT views showing radial, coronal-diagonal and sagittal-diagonal vertex pairs, respectively (white arrows). Contours: green, VTV; magenta, lattice volume (GTV contracted by 20 mm); red, GTV; light blue, CTV; dark blue, PTV.

### Dosimetric analysis: plan quality metrics and VPDR assessment

For each treatment plan, a comprehensive set of dosimetric and geometrical parameters was extracted and compared between Helical Tomotherapy and VMAT FFF delivery. Geometrical characterization of the lattice configuration included the number of vertices (N_vertices_) used for each plan and the center-to-center distance (D_c-c_) between nearest-neighbor vertices. The ratio between the total vertex volume and the gross tumor volume (V_vertices_/V_GTV_) was computed as a measure of the volumetric filling fraction of the lattice within the target. Dose-volume histogram parameters were extracted for the following structures: PTV, VTV, PTV-Lattice (obtained by subtracting the 2 mm-expanded VTV from the PTV, the inter-vertex tissue compartment representing the intended valley-dose region), the GTV, and the External-PTV (normal-tissue surrogate). For the PTV, VTV, PTV-Lattice, and GTV, the following metrics were recorded: D95%, defined as the dose received by at least 95% of the structure volume, used as the primary coverage index; D50%, the median dose delivered to the structure, reflecting the central tendency of the intratumoral dose distribution; D1%, defined as the dose received by the hottest 1% of the structure volume, taken as a surrogate for the near-maximum (peak) dose; V95%Dp=19.95Gy, defined as the percentage volume receiving at least 95% of the prescription dose (Dp = 21.00 Gy, i.e., ≥19.95 Gy), used to assess adequacy of target coverage relative to prescription; and V100%Dp=21.00Gy, defined as the percentage volume receiving at least 100% of the prescription dose, providing a stricter, unmargined coverage metric. For the External-PTV, defined as the body contour excluding the PTV volume, normal-tissue exposure was characterized through D_mean_, the mean dose delivered to the structure, and D1%, the near-maximum dose received by the hottest 1% of its volume; low- and intermediate-dose spread outside the target was further assessed via V5Gy, V10Gy, and V15Gy, defined as the percentage of External-PTV volume receiving at least 5, 10, and 15 Gy, respectively, providing an index of the extent of normal-tissue exposure to low-to-intermediate doses beyond the target boundary. Additionally, the beam-on time and the number of monitor units per fraction (MU/f) were recorded for each plan as measures of delivery efficiency.

### VPDR assessment: multi-method and directionally-resolved analysis

The valley-to-peak dose ratio (VPDR) was evaluated according to three distinct methodologies drawn from the published literature, applied consistently to all treatment plans across both delivery techniques.

The first method followed the pairwise approach described by Prado et al. in 2024 (23). The dose grid exported in DICOM RT-DOSE format was resampled to an isotropic voxel spacing of 1.5 × 1.5 × 1.5 mm using trilinear interpolation. For each vertex, the centroid was computed from the binary mask of the corresponding RT-STRUCT contour projected onto the isotropic dose grid, and the peak dose was defined as D_1%_, the dose received by the hottest 1% of the vertex volume, extracted from the cumulative dose-volume histogram. For each pair of vertices (i, j), the pair peak dose Dp_ij_ was calculated as the arithmetic mean of the respective D_1%_ values. The pair valley dose Dv_ij_ was sampled at the geometric midpoint between the two centroids via trilinear interpolation of the resampled dose volume, using 200 equally spaced points along the connecting line segment. The pairwise VPDR was obtained as ${\text{VPDR}}_{\text{ij}}={\text{Dv}}_{\text{ij}}/{\text{Dp}}_{\text{ij}}$, and the overall VPDR_median_ was reported as the median of all pairwise values, with the interquartile range IQR provided as a measure of dispersion.

The second method followed the DVH-based approach described by Borzov et al. in 2022 (24), in which the VPDR is defined as the ratio ${\text{D}}_{90\%}/{\text{D}}_{10\%}$ computed on the dose distribution within the GTV. In this formulation, D_90%_ represents the dose level below which 90% of the lattice volume is irradiated, approximating the valley dose level, while D_10%_ represents the dose exceeded by only the hottest 10% of the lattice volume, approximating the peak dose level.

The third method followed the definition proposed by Wu et al. (12, 16), in which the valley dose is computed as the mean dose within the coldest 5% of the lattice volume, corresponding to the dose interval between the 95*^th^* and 100*^th^* percentile of the cumulative DVH in descending order, denoted as D_mean(95–100)_, while the peak dose is defined explicitly as the prescription dose D_pres_. The plan VPDR is then obtained as ${\text{VPDR}}_{\text{Wu}}={\text{D}}_{\text{mean}\left(95-100\right)}/{\text{D}}_{\text{p}}$.

Beyond the scalar VPDR metrics described above, (a directionally-resolved analysis was performed using the pairwise framework of Prado et al. (23) to characterize explicitly the dependence of the valley-to-peak dose ratio on the geometric orientation of each vertex pair within the three-dimensional lattice. For each patient, all vertex pairs were classified according to the predominant direction of their connecting vector, identified by comparing the absolute displacements along each anatomical axis. Six directional categories were defined: the three principal anatomical axes (x, left-right; y, anterior-posterior; z, superior-inferior), the radial direction within the transverse plane, the coronal diagonal, and the sagittal diagonal. For each directional category, a direction-specific VPDR was computed as the median of the VPDR_ij_ values of all vertex pairs assigned to that category, yielding six directional indices per patient, VPDR_x_, VPDR_y_, VPDR_z_, VPDR_radial_, VPDR_diag-coronal_, VPDR_diag-sagittal_, in addition to the global VPDR_median_. This directionally-resolved framework makes explicit the anisotropy in peak-valley dose modulation that is an intrinsic geometric consequence of any three-dimensional staggered lattice arrangement, and enables a structured comparison of directional dosimetric performance between Helical Tomotherapy and VMAT FFF delivery.

### Morphological analysis and correlation with directional VPDR

Three-dimensional target morphology was characterized using a custom Python-based (version 3.12) workflow (SimpleITK, scikit-image, scikit-learn). For each GTV, the segmentation mask was resampled onto an isotropic grid (1.5 × 1.5 × 1.5 mm) and principal component analysis (PCA) was applied to the voxel coordinates: the square roots of the covariance matrix eigenvalues provided the semi-axis lengths of the equivalent ellipsoid (a ≥ b ≥ c), from which Aspect Ratio (a/c), Elongation (b/a), Flatness (c/a), and Compactness (c/b) were derived. Surface area was computed via marching cubes, and Wadell Sphericity as the ratio between the surface of an equal-volume sphere and the actual target surface (values approaching 1 for near-spherical shapes).

### Statistical analysis

Descriptive statistics were used to summarize baseline clinical and dosimetric characteristics. Differences in continuous variables (e.g., GTV volume) across clinical categories were evaluated using the Mann-Whitney U test, while associations between categorical variables (e.g., treatment intent and fractionation scheme) were assessed via Fisher’s exact test. Scalar VPDR values obtained with VMAT-LRT and HT-LRT were compared using paired t-tests or Wilcoxon signed-rank tests, as appropriate based on the Shapiro–Wilk test applied to the paired differences; comparisons were stratified by computational method (Wu, D90/D10, and median VPDR), directional category, and anatomical district. Effect size was expressed as Cohen’s d for paired samples (mean paired difference/SD of differences), with values of 0.2, 0.5, and 0.8 indicating small, medium, and large effects, respectively. Within-technique pairwise comparisons were also performed separately for VMAT-LRT and HT-LRT, using the Wilcoxon signed-rank test on all cases with evaluable values for both metrics under comparison. Two independent hypothesis families were defined and corrected separately using the Bonferroni method: (i) scalar method comparisons among VPDR_Wu_, VPDR_D90/D10_, and VPDR_median_ (k = 3, α_corr_ = 0.0167), and (ii) directional pairwise comparisons among the six geometric categories (radial, Z, X, coronal-diagonal, sagittal-diagonal, Y; k = 15, α_corr_ = 0.0033). Spearman’s rank correlation coefficients (*ρ*) were calculated to assess monotonic relationships between morphological or target volume features and radiotherapy parameters, including the three scalar estimators and the six directional pairwise categories. For all analyses, statistical significance was set at p< 0.05, or at the Bonferroni-corrected threshold where applicable.

## Results

A total of 22 patients were included, with a median of 5 lattice vertices per case (range 2–21), reflecting the wide variability in target volume (median GTV 751.9 cc, range 380.2–4927.7 cc).

Baseline demographic, histological, and treatment characteristics are summarized in **Table 1**. Statistical analysis revealed that GTV volumes tended to be larger in patients treated with neoadjuvant intent compared to palliative cases (*p* = 0.052). Furthermore, treatment intent significantly influenced the choice of fractionation scheme (*p* < 0.001) and total delivered dose, with neoadjuvant plans achieving higher BED_10_ values (*p* < 0.001). Target volume significantly correlated with the number of fractions (*ρ* = 0.667, *p* < 0.001), total physical dose (*ρ* = 0.600, *p* < 0.001), and overall BED_10_ (*ρ* = 0.602, *p* < 0.01).

**Table 1:** Baseline Patient Demographics, Histological Features, Radiotherapy Parameters, and Statistical Correlations (n = 22).

Parameter	Subcategory/Metric	Value, n (%)	GTV volume (cc) median (Min–Max)	Statistical test/association
Anatomical District	Extremity	9 (40.9%)	—	—
	Thorax/Head & Neck	7 (31.8%)	—	—
	Abdomen/Pelvis	6 (27.3%)	—	—
Histological Types	Liposarcoma (all subtypes)	9 (40.9%)	—	—
	Other Soft Tissue Sarcomas	6 (27.3%)	—	—
	Mediastinal/Thoracic Carcinomas (SCLC, NSCLC, Adeno)	4 (18.2%)	—	—
	Gynecological Carcinoma/Sarcoma	2 (9.1%)	—	—
	Melanoma	1 (4.5%)	—	—
Histological Grade	High Grade (G3/High/Dedifferentiated)	14 (63.6%)	—	—
	Low Grade/Intermediate Grade (G1/G2)	7 (31.8%)	—	—
	Not Defined (NDD)	1 (4.5%)	—	—
Clinical Presentation – Local Infiltration	Yes	12 (54.5%)	—	—
	No	10 (45.5%)	—	—
Clinical Presentation – Recurrent Disease	Yes (≥2 Relapses)	2 (9.1%)	—	—
	No (Primary/1st Relapse)	20 (90.9%)	—	—
Original Treatment Intent	Neoadjuvant Radiotherapy	11 (50.0%)	1003.4 (611.9 – 4927.7)	1. GTV Volume vs. Intent: Mann-Whitney U = 90.0, p = 0.052 (Neoadjuvant vs. Palliative Cohort) 2. Intent vs. Scheme/Dose: • Fisher’s Exact Test: p = 0.0003 (Standard 50Gy/25fx vs. Hypo/Short/Single-fx) • BED_10_ Dose Comparison: Mann-Whitney U = 113.5, p = 0.0002 (Median BED_10_: 60.0 Gy [Neoadjuvant] vs. 30.0 Gy [Palliative])
	Palliative Radiotherapy (Total)	11 (50.0%)	572.0 (380.2 – 3119.0)	
	• Symptomatic/General Palliative	9 (40.9%)	572.0 (380.2 – 3119.0)	
	• Cytoreductive-only Intent	1 (4.5%)	1404.7	
	• Acute Hemostatic Intent	1 (4.5%)	380.2	
Original Fractionation Schemes	50 Gy in 25 fractions [Median BED_10_: 60.0 Gy]	11 (50.0%)	975.2 (611.9 – 4451.6)	Spearman Rank Correlation (GTV Volume vs. RT Parameters): • GTV vs. No. of Fractions: ρ = 0.667, p = 0.0007 • GTV vs. Total Dose: ρ = 0.600, p = 0.0032 • GTV vs. BED_10_: ρ = 0.602, p = 0.0030
	20–30 Gy in 4–10 fractions [Median BED_10_: 30.0 Gy]	6 (27.3%)	520.4 (394.3 – 701.8)	
	40–54 Gy in 16–27 fractions [Median BED_10_: 53.1 Gy]	4 (18.2%)	2684.1 (1404.7 – 4927.7)	
	8 Gy in 1 fraction [BED_10_: 14.4 Gy]	1 (4.5%)	380.2	

BED_10_, Biologically Effective Dose calculated using an *α*/*β* ratio of 10 Gy; EBRT, External Beam Radiotherapy; GTV, Gross Tumor Volume; ND, Not Defined/Not Determined; NSCLC, Non-Small-Cell Lung Cancer; SCLC, Small-Cell Lung Cancer.

The median vertex-to-GTV volume ratio (V_vert_/V_GTV_) was 0.2% (range 0.1-0.3%) consistently low across all cases (12, 20). This low filling fraction is a direct consequence of the conservative geometric parameters adopted (2 cm GTV contraction, 5 cm Dc-c, 1 cm vertex diameter). The geometric characteristics of the lattice array across the study cohort are summarized in **Supplementary Table S1** (**Supplementary**), and the directional classification framework is illustrated in **Figure 1** and **Figure 2**. The directional analysis of centre-to-centre distances revealed a systematic and geometrically coherent anisotropy inherent to the staggered triangular lattice arrangement (**Figure 2A**). The radial intra-plane distance (D_c-c_(Radial) in **Figures 1**, **2B**) was highly consistent across the cohort (median 50.4 mm, range 35.4–51.3 mm; **Supplementary Table S1**), with only two cases deviating from the nominal spacing due to patient-specific adaptation of the lattice to smaller target dimensions (Cases 17 and 18, radial D_c-c_ 40.6 mm and 35.4 mm, respectively). The inter-plane axial distance (Z, superior–inferior direction, **Figure 1**, magenta arrow) was equally stable (median 54.0 mm, range 53.8–54.3 mm), marginally exceeding the radial spacing as expected from the geometry of the staggered arrangement. In contrast, distances along the lateral (X) and anterior-posterior (Y) directions (**Figure 1**, green and orange arrows, respectively) were substantially larger (medians 71.2 mm and 71.0 mm, respectively), reflecting the inter-column spacing of the triangular grid projected onto those anatomical axes. Coronal and sagittal diagonal distances, arising from the combination of in-plane radial displacement and inter-plane axial offset (**Figures 2C**, **2D**, respectively) were systematically shorter than the radial spacing (medians 44.8 mm and 44.7 mm), corresponding to the projected inter-plane nearest-neighbour separation. Importantly, not all directional categories were identifiable in every case: the X and Y directions in particular required a minimum number of vertices and a specific spatial arrangement to yield classifiable pairs, and were absent in the majority of cases with fewer than five vertices (**Supplementary Table S1**). This patient- and direction-specific sampling heterogeneity, a structural consequence of adapting the lattice geometry to individual tumour morphology, provides a direct quantitative rationale for the directionally-weighted VPDR framework proposed in this study, in which each directional contribution is weighted proportionally to the number of vertex pairs available in that direction.

VPDR values across all three computational methods (**Figure 3**) and all directional categories (**Figure 4**) are reported in **Supplementary Table S2**, with the corresponding inter-vertex dose profiles illustrated in **Figure 5**. Across the full cohort (n = 22), HT-LRT achieved significantly lower median VPDR than VMAT-LRT by all three scalar methods, with large Cohen’s (d) effect sizes: VPDR_Wu_ 0.29 vs 0.37 (d = 1.59, p< 0.001), VPDR_D90/D10_ 0.31 vs 0.42 (d = 2.07, p< 0.001), and VPDR_median_ 0.46 vs 0.54 (d = 1.08, p< 0.001). The superiority of HT-LRT was confirmed across all three anatomical subgroups (Abdomen, Extremity, Thorax), with the largest effect sizes observed in the Abdomen group (VPDR_Wu_, d = 3.71, p< 0.001; VPDR_D90/D10_, d = 4.78, p< 0.001; VPDR_median_, d = 1.94, p< 0.05), suggesting that the dosimetric advantage of HT-LRT is most pronounced for deep-seated abdominal targets.

**Figure 3:**
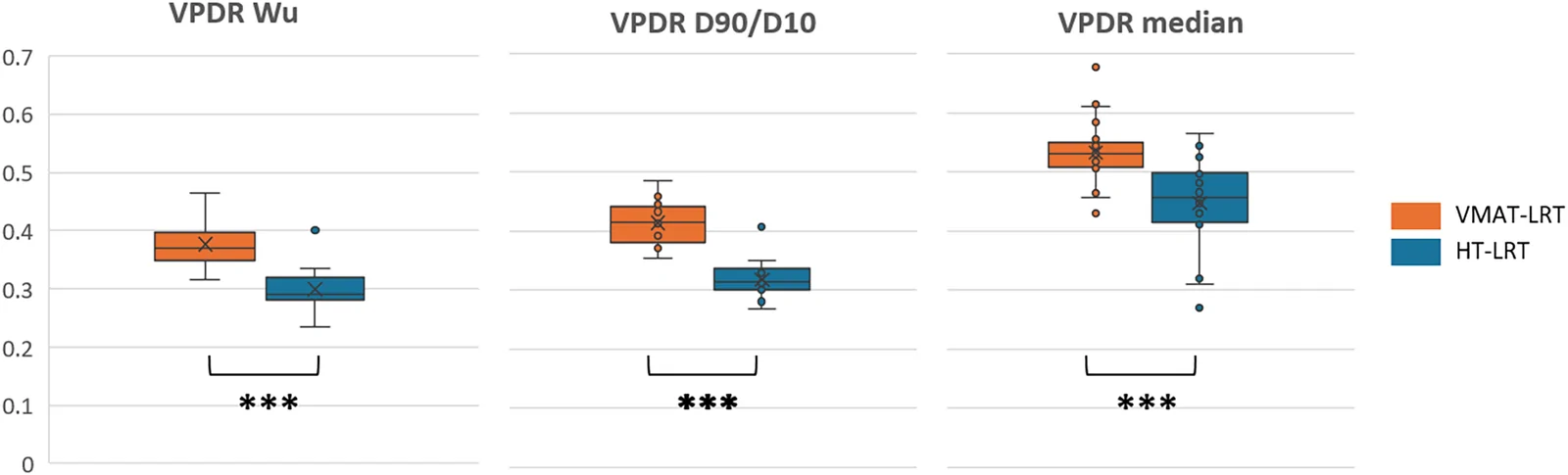
Comparison of scalar VPDR values distributions between VMAT-LRT (orange) and HT-LRT (blue) computed using three different methods: VPDR_Wu_ (left), VPDR_D90/D10_ (center), and VPDR_median_ (right). Box plots display IQR, median (horizontal lines), mean (×) and dots for individual patients. Statistical significance of paired comparisons between techniques is indicated below each panel (***p< 0.001, **p< 0.01, *p< 0.05; ns, not significant; paired t-test or Wilcoxon signed-rank test as appropriate; **Supplementary Table S2** of **Supplementary**).

**Figure 4:**
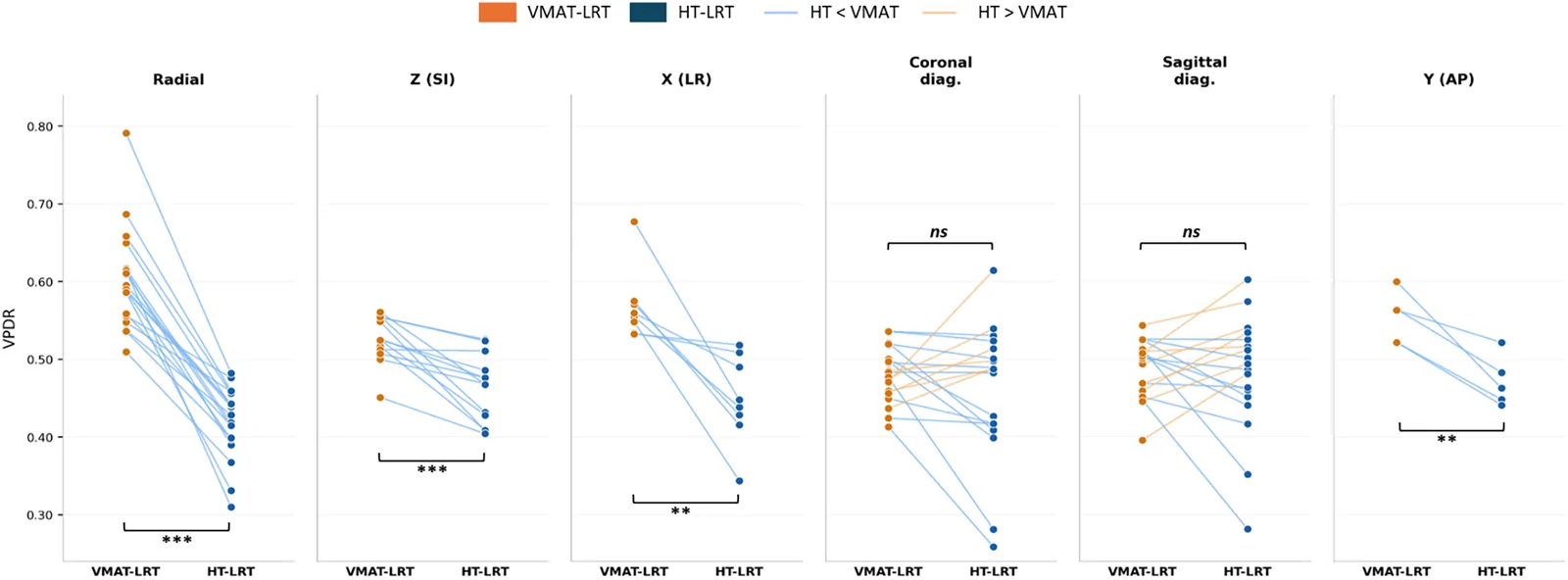
Paired directional VPDR comparison across six different geometric directions. Each panel displays paired per-patient VPDR values for VMAT-LRT (orange dots) and HT-LRT (blue dots) connected by individual lines, stratified by directional category: Radial, Z (superior–inferior), X (left–right), coronal diagonal, sagittal diagonal, and Y (anterior–posterior). Line colors encode the direction of the within-patient difference: blue lines indicate cases where HT-LRT achieved lower VPDR than VMAT-LRT (HT< VMAT), and orange lines indicate the opposite case (HT > VMAT). Statistical significance of paired comparisons is indicated below each panel (***p< 0.001, **p< 0.01, *p< 0.05; ns, not significant; paired t-test or Wilcoxon signed-rank test as appropriate; **Supplementary Table S2** of **Supplementary**).

**Figure 5:**
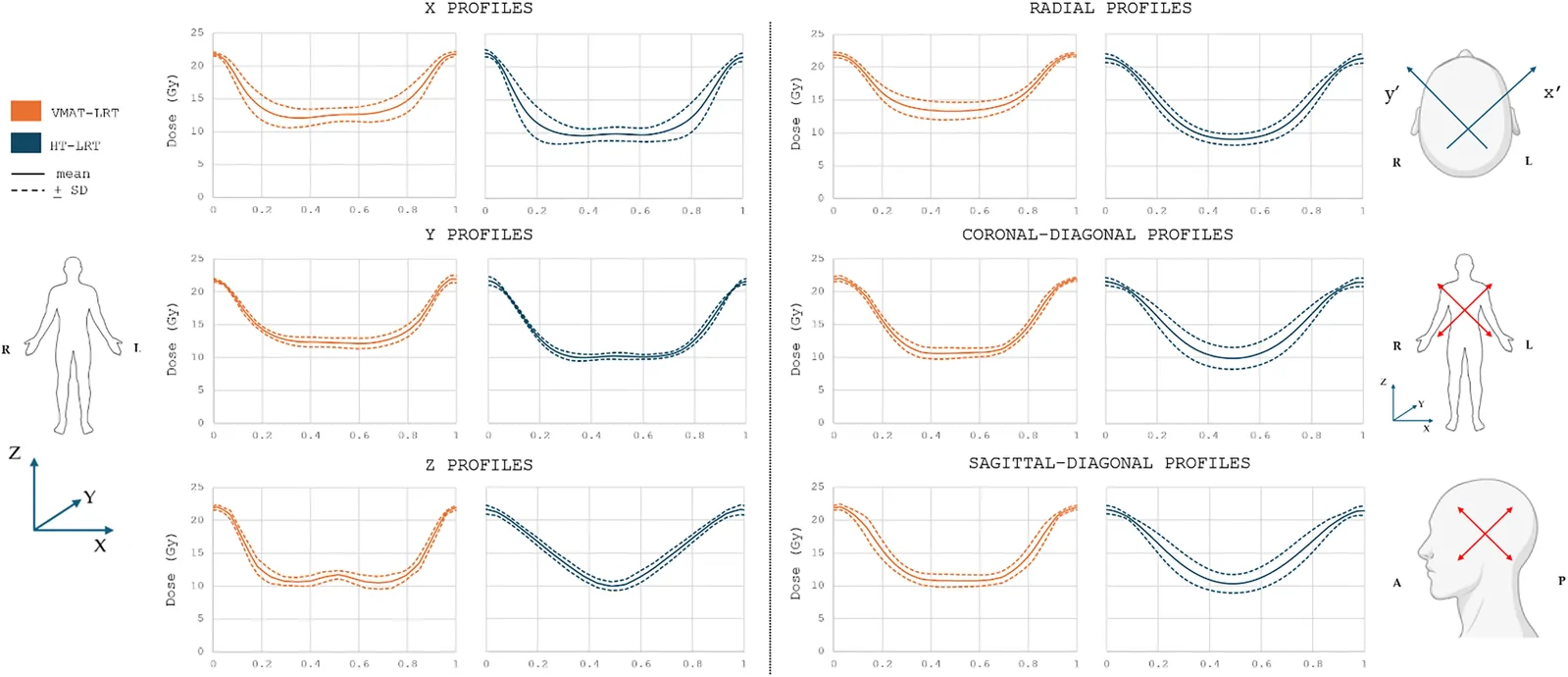
Directionally-resolved inter-vertex dose profiles for VMAT-LRT and HT-LRT across the study cohort. Mean dose profiles (solid lines) with ± 1 SD (dashed lines) for VMAT-LRT (orange) and HT-LRT (blue), plotted as a function of normalized position along the inter-vertex connecting segment (0 = centroid of vertex i, 1 = centroid of vertex j). Profiles are stratified by direction (see **Figure 2**): X (left–right), Y (anterior–posterior), Z (superior–inferior), radial (in-plane oblique), coronal diagonal, and sagittal diagonal. Anatomical body diagrams and corresponding directional indicators (red arrows) are provided alongside each row to illustrate the spatial orientation of the connecting vector within the patient reference frame. The x-axis represents the normalized curvilinear distance between vertex centroids; the y-axis reports absolute dose (Gy). Peak doses at positions 0 and 1 correspond to the D_1%_ of the respective vertices; the dose minimum at the midpoint (position 0.5) corresponds to the valley dose Dv_ij_ used in the pairwise VPDR computation according to the method of Prado et al. (23).

Directional decomposition of the pairwise VPDR (**Figure 4**) revealed a consistent and technique-specific interpretable pattern, visible in the dose profiles of **Figure 5**. HT-LRT produced significantly lower VPDR than VMAT-LRT in the radial (0.43 vs 0.59, d = 2.84, p< 0.001), Z superior–inferior (0.47 vs 0.53, d = 1.45, p< 0.001), X left–right (0.44 vs 0.56, d = 1.52, p = 0.004), and Y anterior–posterior (0.46 vs 0.56, d = 2.41, p = 0.006) directions. Strikingly, no significant difference was observed between the two techniques in either the coronal diagonal (0.49 vs 0.48, d = 0.24, p = 0.321) or the sagittal diagonal direction (0.49 vs 0.50, d = 0.12, p = 0.625), indicating that the dosimetric advantage of HT-LRT is direction-selective and does not extend uniformly to inter-plane oblique trajectories. This finding was robust across anatomical subgroups since coronal and sagittal diagonal VPDR remained non-significant into extremity, and thoracic subgroups. Taken together, these findings demonstrate that scalar VPDR metrics, by averaging across geometrically heterogeneous directions, obscure a systematic and clinically relevant directional structure in the peak-valley dose modulation pattern that may differ between delivery techniques.

Within-technique analysis of scalar and directional VPDR variability revealed a geometrically coherent and technique-specific pattern (**Supplementary Table S3**, **Supplementary**). Among scalar methods, all three pairwise comparisons (Wu vs D90/D10, Wu vs Median, D90/D10 vs Median) yielded statistically significant differences after Bonferroni correction (α_corr_ = 0.0167) for both techniques (Cohen’s d ranging from −1.64 to −2.46). The consistently negative sign of Cohen’s d reflects a systematic ordering VPDR_Wu<_ VPDR_D90/D10<_ VPDR_median_ (p< 0.001), replicating on a larger paired cohort the observations of Prado et al. (23). Notably, HT-LRT displayed a pattern analogous to that observed for VMAT-LRT, with a smaller effect size for the Wu vs D90/D10 comparison (d = −1.64 vs −2.10, respectively).

Regarding directional comparisons (α_corr_ = 0.0033, k = 15), in VMAT-LRT the radial VPDR was significantly higher than both the coronal diagonal VPDR (p< 0.0001, d = +1.55) and the sagittal diagonal VPDR (p< 0.0001, d = +1.43), confirming that intra-plane vertex pairs along the radial direction systematically exhibit warmer valley doses than inter-plane oblique pairs. The absence of significant differences across the remaining directional comparisons confirms the overall consistency of the peak-valley dose ratio throughout the three-dimensional lattice volume, underscoring the importance of directional VPDR assessment for an adequate evaluation of spatially fractionated treatments characterized by uniform peak and valley doses across directions.

Similarly, in HT-LRT the only significant difference was observed between radial and sagittal diagonal VPDR (p< 0.001, d = −1.69). Specifically, HT-LRT achieves a significantly lower, and therefore more favorable, radial VPDR relative to the sagittal diagonal direction. This represents a direct inversion of the directional hierarchy observed with VMAT-LRT: the direction in which VMAT-LRT exhibits its greatest dosimetric limitation is precisely the direction in which HT-LRT achieves its best performance, confirming the existence of an inter-technique directional anisotropy in the VPDR pattern. The absence of significant differences across the remaining directional comparisons confirms the overall consistency of the peak-valley dose ratio throughout the three-dimensional volume. The fact that the directional structure of VPDR is robustly heterogeneous only for the radial vs diagonal pair, and that this difference is both geometrically motivated by the staggered triangular lattice geometry and technique-specific in the direction of its effect, suggests that median VPDR aggregates dosimetrically homogeneous directions in the majority of cases, providing a stable and representative estimate of the overall peak-valley dose modulation pattern. The directional decomposition simultaneously retains the ability to identify the specific geometric axis along which each technique concentrates its dosimetric limitation, information that scalar summary metrics are inherently unable to capture.

Dosimetric parameters for all relevant structures are reported in **Table 2** and DVH curves in **Figure 6**. Regarding vertex coverage, both techniques achieved clinically adequate dose delivery to the VTV, with no significant difference in D95% (median values, 20.03 Gy VMAT-LRT vs 20.23 Gy HT-LRT, p = 0.116). However, HT-LRT yielded significantly higher V95%Dp (98.77% vs 96.07%, p = 0.032), while VMAT-LRT produced modestly higher D50% (21.21 vs 21.00 Gy, p< 0.001) and D1% (22.66 vs 22.15 Gy, p = 0.014), reflecting a slight tendency toward hotspot concentration within individual vertices.

**Table 2:** DVH-based dosimetric parameters for VMAT-LRT and HT-LRT (n = 22 paired cases), reported as median (min–max).

Structure	Dosimetric parameter	VMAT-LRT	HT-LRT	p-value	Sig.
		Median (min–max)			
PTV	D95% (Gy)	8.55 (7.38-10.51)	6.64 (5.50-8.63)	<0.001	***
	D50% (Gy)	10.62 (9.23-11.64)	7.28 (6.61-9.08)	<0.001	***
	D1% (Gy)	15.30 (12.83-16.22)	16.38 (14.29-18.07)	<0.001	***
	V95%Dp=19.95Gy (%vol)	0.19 (0.09-0.31)	0.18 (0.10-0.31)	0.644	ns
	V100%Dp=21.00Gy (%vol)	0.07 (0.03-0.15)	0.06 (0.03-0.12)	0.038	*
VTV	D95% (Gy)	20.03 (19.86-20.46)	20.23 (19.73-20.80)	0.116	ns
	D50% (Gy)	21.21 (20.70-21.64)	21.00 (20.89-22.14)	<0.001	***
	D1% (Gy)	22.66 (21.62-23.65)	22.15 (21.28-23.91)	0.014	*
	V95%Dp=19.95Gy (%vol)	96.07 (93.57-98.62)	98.77 (91.46-100.00)	0.032	*
	V100%Dp=21.00Gy (%vol)	60.36 (25.12-74.45)	50.10 (34.83-89.38)	<0.001	***
PTV-Lattice*	D95% (Gy)	8.55 (7.37-10.50)	6.64 (5.50-8.63)	<0.001	***
	D50% (Gy)	10.61 (9.23-11.63)	7.28 (6.60-9.08)	<0.001	***
	D1% (Gy)	14.86 (12.73-15.84)	15.82 (13.25-17.80)	0.001	***
	V95%Dp=19.95Gy (%vol)	0.03 (0.00-0.09)	0.03 (0.00-0.18)	0.859	ns
	V100%Dp=21.00Gy (%vol)	0.00 (0.00-0.02)	0.00 (0.00-0.01)	0.851	ns
External-PTV	V5Gy (%vol)	5.10 (0.06-14.52)	2.29 (0.00-16.55)	0.008	**
	V10Gy (%vol)	0.03 (0.00-0.37)	0.00 (0.00-0.03)	<0.001	***
	V15Gy (%vol)	0.00 (0.00-0.02)	0.00 (0.00-0.02)	1.000	ns
	Dmean (Gy)	1.08 (0.60-2.16)	0.93 (0.51-2.68)	0.519	ns
	D1% (Gy)	7.62 (4.08-9.18)	6.03 (3.23-8.23)	0.001	***
GTV	D95% (Gy)	9.86 (8.08-10.88)	6.91 (6.28-8.82)	<0.001	***
	D50% (Gy)	11.03 (9.24-11.82)	7.45 (6.73-9.12)	<0.001	***
	D1% (Gy)	17.28 (14.88-18.86)	18.51 (16.24-19.67)	0.007	**
	V95%Dp=19.95Gy (%vol)	0.37 (0.19-0.67)	0.39 (0.15-0.77)	0.431	ns
	V100%Dp=21.00Gy (%vol)	0.15 (0.04-0.30)	0.11 (0.04-0.28)	0.116	ns
Beam on time (s)		389.00 (227.00-759.00)	961.50 (519.00-2358.40)	<0.001	***
Monitor Unit per Fraction (MU/f)		1969.90 (1627.00-3153.00)	13813.80 (7456.00-33882.00)	<0.001	***

***p< 0.001, **p< 0.01, *p< 0.05; ns, not significant.

Dp, prescription dose (21 Gy in 3 fractions); V95%Dp and V100%Dp, percentage volume receiving ≥ 95% and ≥ 100% of the prescription dose, respectively; Dx%, dose received by x% of the structure volume; Vx Gy, percentage volume receiving ≥ x Gy. PTV, planning target volume (whole tumor); VTV, vertex target volume (union of individual lattice vertex PTVs); PTV-Lattice, PTV minus the 2mm expanded VTV region (inter-vertex tissue compartment, representing the intended valley dose region); External-PTV, body contour excluding PTV (normal tissue surrogate); GTV, gross tumor volume. Beam-on time and monitor units per fraction (MU/f) are reported as treatment efficiency parameters.

Wilcoxon signed-rank test or paired t-test (Shapiro–Wilk test on paired differences).

**Figure 6:**
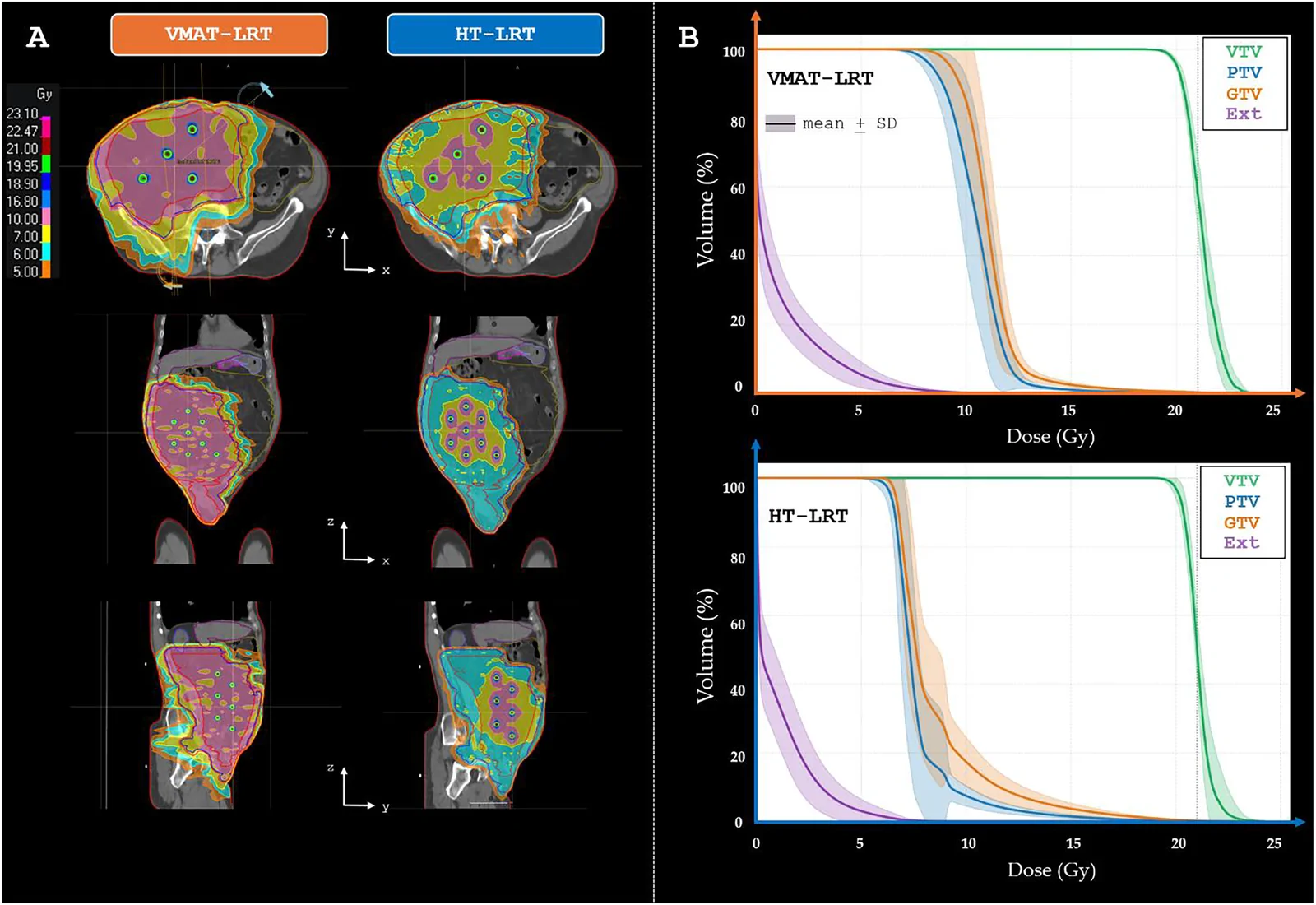
Comparative dose distributions and mean DVH curves for VMAT-LRT and HT-LRT. **(A)** Representative multiplanar dose distributions for a large abdominal sarcoma case, shown in axial (top), coronal (middle), and sagittal (bottom) planes for VMAT-LRT (left) and HT-LRT (right). Isodose lines are displayed from 5.00 Gy to 23.10 Gy. Contours: red, GTV; orange, PTV; magenta, Lattice Volume; green circles, individual lattice vertices (VTV). **(B)** Mean DVHs (solid lines) with ±1 SD (shaded areas) for the four primary dosimetric structures, VTV (green), PTV (blue/orange), GTV (orange/blue), and External-PTV (purple), across the cohort (n = 22) for VMAT-LRT (top) and HT-LRT (bottom); prescription dose (21 Gy, vertical dashed line).

The most clinically relevant differences between techniques emerged in the inter-vertex tissue compartments. In both the PTV-Lattice (representing the PTV volume excluding an uniform 2 mm expanded VTV) and the global GTV, VMAT-LRT delivered significantly higher doses at D95% (PTV-Lattice: 8.55 vs 6.64 Gy, p< 0.001; GTV: 9.86 vs 6.91, p< 0.001) and D50% (PTV-Lattice: 10.61 vs 7.28 Gy, p< 0.001; GTV: 11.03 vs 7.45 Gy, p< 0.001) than HT-LRT, reflecting substantially warmer valley doses with VMAT-LRT. Conversely, HT-LRT achieved higher D1% in both structures (PTV-Lattice: 15.82 vs 14.86 Gy, p = 0.001; GTV: 18.51 vs 17.28 Gy, p = 0.007), consistent with a steeper dose gradient between peaks and valleys, a finding directly concordant with the lower VPDR values observed for HT-LRT across all computational methods. The volumetric coverage of the prescription isodose within the GTV (V95%Dp and V100%Dp) did not differ significantly between techniques (p = 0.431 and p = 0.116, respectively), confirming that the dosimetric superiority of HT-LRT in valley dose suppression was not achieved at the expense of overall GTV coverage.

Normal tissue sparing, assessed on the External-PTV structure, was significantly better for HT-LRT compared to VMAT-LRT for low-to-intermediate dose levels: V5Gy was 2.29% vs 5.10% (p = 0.008) and V10Gy was effectively zero with HT-LRT vs 0.03% with VMAT-LRT (p< 0.001). The near-maximum dose to the external volume (D1%) was also significantly lower with HT-LRT (6.03 vs 7.62 Gy, p = 0.001), while mean dose did not differ significantly between techniques, suggesting that the normal tissue advantage of HT-LRT is concentrated at the lower end of the dose spectrum. These differences in peripheral dose distribution are consistent with the rotational and highly modulated delivery characteristic of Helical Tomotherapy.

Finally, a substantial difference in treatment efficiency was observed. VMAT-LRT required a median beam-on time of 389 seconds per fraction (range 227–759 s) and a median of 1969.9 MU per fraction (range 1627–3153 MU/f), compared to 961.5 seconds (range 519–2358 s, p< 0.001) and 13813.8 MU per fraction (range 7456–33882 MU/f, p< 0.001) for HT-LRT. The approximately 2.5-fold longer beam-on time and 7-fold higher monitor unit demand of HT-LRT represent relevant practical considerations in the clinical implementation of lattice radiotherapy, particularly with respect to intrafraction motion management and machine workload.

The three-dimensional morphological characterization of the study cohort is summarized in **Supplementary Table S4** of **Supplementary**. Target volumes were heterogeneous, with a median GTV of 782 cc (range 397–5032 cc) and a wide distribution of shape indices across anatomical subgroups. Abdomen cases showed the largest volumes and the most irregular geometries, while Thorax cases were characterized by smaller, more compact targets. Across the full cohort, the median Aspect Ratio (AR, a/c ratio) of 1.77 (range 1.17–3.27) and Wadell Sphericity of 0.66 (range 0.53–0.79) indicated a predominantly non-spherical geometry. Extremity tumors exhibited the widest range of Aspect Ratios (1.48–3.27), reflecting the anatomical variability of soft-tissue sarcomas in this region.

To assess whether intra-technique VPDR variability was systematically associated with target morphology, Spearman correlation analysis was performed between morphological features and directional VPDR values for both techniques (**Supplementary Table S5**; **Figure 7**). For scalar VPDR metrics, correlations with morphological features were generally weak and technique-dependent. In VMAT-LRT, VPDR_D90/D10_ showed a moderate positive correlation with GTV volume (ρ = 0.48, p< 0.05; **Figure 7**, top center), suggesting that larger tumors tend to produce warmer valley doses when planned with VMAT. Wadell Sphericity correlated positively with both VPDR_Wu_ (ρ = 0.51, p< 0.05) and VPDR_D90/D10_ (ρ = 0.49, p< 0.05), indicating that more spherical targets are associated with higher scalar VPDR under arc-based delivery. In contrast, no significant correlation between scalar VPDR and GTV volume was observed for HT-LRT across any of the three methods (ρ = 0.04–0.36, all ns; **Figure 7**, top row), indicating that HT-LRT VPDR is substantially less sensitive to tumor volume than VMAT-LRT. VPDR_median_ showed no significant correlation with volume in either technique (VMAT ρ = 0.05; HT-LRT ρ = 0.36), further supporting the interpretation that pairwise midpoint methods capture a different dosimetric dimension than DVH-derived scalar metrics.

**Figure 7:**
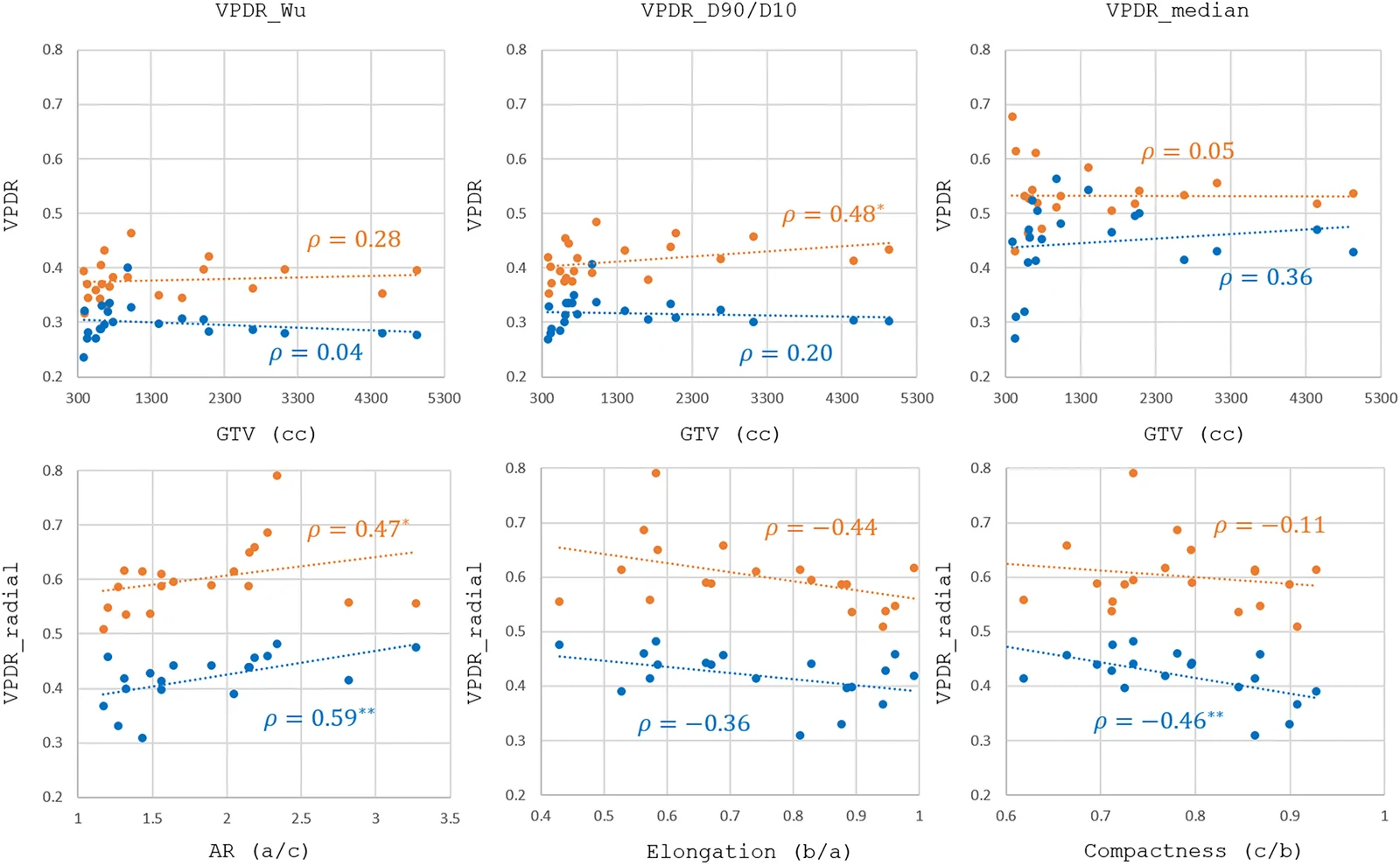
Spearman correlations between morphological features and VPDR for VMAT-LRT (orange) and HT-LRT (blue). Each panel displays per-patient scatter plots with Spearman’s ρ coefficients and fitted regression lines (dotted) for each technique. Top row: scalar VPDR metrics (VPDR_Wu_, VPDR_D90/D10_, VPDR_median_) plotted against GTV volume (cc). Bottom row: radial pairwise VPDR (VPDR_radial_) plotted against Aspect Ratio a/c (left), Elongation b/a (center), and Compactness c/b (right). Significance: **p< 0.01, *p< 0.05; unlabelled coefficients are non-significant.

Directional analysis revealed more informative and geometrically coherent morphology–VPDR relationships. The radial VPDR, corresponding to the primary intra-plane vertex spacing direction, showed a significant positive correlation with Aspect Ratio in both techniques (VMAT-LRT ρ = 0.47, p< 0.05; HT-LRT ρ = 0.59, p< 0.01; **Figure 7**, bottom left), indicating that geometrically elongated targets systematically produce warmer radial valleys regardless of delivery modality. This is physically consistent with a dose summation effect intrinsic to the staggered triangular lattice geometry: by construction, each inter-plane vertex is positioned above the centroid of the triangle formed by the three nearest in-plane vertices, placing it equidistant from, and therefore depositing dose symmetrically into, the three surrounding radial valley regions. In elongated targets accommodating multiple vertex planes along the major axis, radial valley points receive cumulative dose contributions from inter-plane vertices in both the superior and inferior directions simultaneously, systematically elevating valley doses and consequently increasing radial VPDR values. This geometric dose summation effect is absent or minimal in compact, near-spherical targets with fewer vertex planes. Compactness (c/b) was additionally negatively associated with radial VPDR specifically in HT-LRT (ρ = −0.46, p< 0.05; **Figure 7**, bottom right), with more compact targets achieving lower radial valley doses under helical delivery, a relationship that did not reach significance with VMAT-LRT (ρ = −0.11, ns), suggesting a technique-specific sensitivity to the minor-axis geometry of the target.

In VMAT-LRT, the coronal diagonal VPDR showed moderate positive correlations with volume (ρ = 0.55, p< 0.05), semi-axis b (ρ = 0.49, p< 0.05), and Wadell Sphericity (ρ = 0.48, p< 0.05), indicating that larger and more spherical targets tend to exhibit warmer coronal diagonal valley doses under arc-based delivery. This pattern was not replicated in HT-LRT (all ns), suggesting that the sensitivity of coronal diagonal VPDR to target morphology is technique-specific and may reflect geometric constraints inherent to the arc arrangement of VMAT-LRT.

The strongest morphological associations emerged in the Y (anterior–posterior) direction, where large negative correlations were observed with multiple size descriptors in both techniques: VMAT-LRT VPDR_Y correlated negatively with volume (ρ = −0.60, p< 0.05), semi-axis a (ρ = −0.70, p< 0.05), and semi-axes b and c (ρ = −0.60, p< 0.05 each), while HT-LRT showed associations of similar or greater magnitude (volume ρ = −0.90, p< 0.05; semi-axes b and c ρ = −0.90, p< 0.05; **Supplementary Table S5**), indicating that larger tumors tend to achieve lower, and therefore more favorable, VPDR values in the AP direction with both techniques. Conversely, Wadell Sphericity showed a strong positive correlation with VPDR_Y under VMAT-LRT (ρ = 0.90, p< 0.05; **Supplementary Table S5**), implying that more spherical targets are associated with warmer AP valleys specifically with arc-based delivery, likely reflecting the predominantly coplanar geometry of VMAT arc arrangements, which provides less angular diversity along the AP axis for compact, near-spherical targets. This association was not significant in HT-LRT (ρ = 0.60, ns), consistent with the more isotropic angular sampling achieved by helical rotational delivery. These results must however be interpreted with considerable caution, as the Y direction was evaluable in only n = 5 cases across the cohort due to the geometric constraints of the lattice arrangement; the observed correlations, while numerically large, are based on a sample size that provides insufficient statistical power for robust inference and should be regarded as exploratory. Taken together, these findings demonstrate that morphological features exert direction-selective and technique-specific influences on the spatial structure of the peak-valley dose pattern in LRT, further underscoring the added interpretive value of the directionally-resolved VPDR framework over scalar summary metrics.

## Discussion

LRT is a novel SFRT modality delivering ablative doses to discrete high-dose spherical subvolumes (vertices) embedded within a tumor volume, alternating with low-dose valley regions, with potential to promote immune-mediated bystander and abscopal effects in bulky, radioresistant neoplasms. While VMAT-LRT currently represents the most widely adopted delivery approach in the LRT literature (20), Helical Tomotherapy-based LRT may offer enhanced spatial dose modulation for large-volume targets.

Prior investigations of Helical Tomotherapy for LRT have been limited to phantom-based dosimetric settings. In 2025, Seol et al. (21) quantitatively analyzed for the first time the impact of vertex size and center-to-center spacing on the valley-to-peak dose ratio (VPDR) in LRT delivered via Helical Tomotherapy, demonstrating that VPDR decreases with increasing vertex size and spacing, with systematically lower values observed along the superior-inferior axis, findings that provide a crucial foundation for optimizing LRT planning on the Tomotherapy platform. Subsequently, in 2026, Sihawong et al. (22) performed a phantom-based dosimetric comparison of helical and fixed-beam Tomotherapy for SFRT using both GRID and lattice target designs, demonstrating that TomoHelical delivery combined with lattice configurations provided superior dose modulation, higher peak-to-valley dose ratios (PVDR), and improved normal tissue sparing, albeit at longer delivery times; all plans satisfied prescription and delivery accuracy requirements, with gamma passing rates exceeding 95%.

Building on these phantom-based findings, this study extends the investigation to a patient-specific clinical setting; Specifically, the present work therefore pursues a dual objective: to characterize the relationships among established VPDR computational methods and three-dimensional target morphological features in a uniformly planned cohort, and to provide the first paired retrospective dosimetric comparison between HT-LRT and VMAT-LRT across scalar, directional, and DVH-based endpoints. A staggered triangular lattice arrangement was employed in this study according to previously published experiences (4, 12, 18, 23).

HT-LRT demonstrated consistent and clinically meaningful dosimetric superiority over VMAT-LRT across all three scalar VPDR methods, with large effect sizes throughout and the most pronounced advantage observed in abdominal targets. These findings extend the observations of Prado et al. (23), who reported VPDR values ranging from 0.16 to 0.28 in a single-fraction VMAT-LRT cohort of 10 patients, noting significant differences among the three computational methods and calling for unified definitions of valley and peak doses. In the present multi-fraction cohort, higher absolute VPDR values were observed for both techniques, consistent with the different prescription scheme and fractionation, but the ordering among methods and the direction of inter-technique differences are fully concordant with existing literature. Specifically, a systematic ordering VPDR_Wu_< VPDR_D90/D10<_ VPDR_median_ was confirmed in both techniques, with all pairwise scalar comparisons reaching significance after Bonferroni correction and large effect sizes (Cohen’s d ranging from −1.64 to −2.46), replicating and extending on a larger paired cohort the findings of Prado et al. (23). The present study translates the phantom-based observations of Seol et al. (21) and Sihawong et al. (22) into a clinical paired cohort, confirming that the dosimetric advantages of helical delivery are maintained and quantifiable in patient-specific anatomical conditions across a wide range of target volumes and anatomical sites.

A central finding of this study is that the dosimetric advantage of HT-LRT over VMAT-LRT is direction-selective rather than uniform across the three-dimensional lattice volume. Significant differences have been observed in the radial, Z, X, and Y directions, while the coronal and sagittal diagonal directions showed no significant inter-technique difference, and in the Extremity subgroup the trend nominally reversed for diagonal components. This directional structure cannot be detected by scalar VPDR metrics and represents the primary methodological contribution of the directionally-resolved framework proposed in this work. The absence of HT-LRT advantage in diagonal inter-plane directions is consistent with the delivery geometry of Helical Tomotherapy: the continuous rotational delivery with couch translation along the SI axis generates dose gradients that are optimally expressed in the axial and radial directions, while inter-plane oblique trajectories sample a dose domain that is less directly modulated by the helical beam pattern. This interpretation aligns with Seol et al. (21), who reported consistently lower VPDR values in the SI direction for Helical Tomotherapy across all phantom configurations, attributing this anisotropy to the delivery characteristics of helical rotation. The present data extend this observation to the full directional decomposition of the staggered triangular lattice geometry, revealing that helical delivery concentrates its advantage primarily in the intra-plane radial direction, where continuous gantry rotation provides optimal angular sampling, a finding entirely invisible to scalar summary metrics.

The within-technique analysis revealed that VMAT-LRT and HT-LRT exhibit diametrically opposite directional VPDR hierarchies: VMAT-LRT shows its primary dosimetric limitation in the radial direction, with radial VPDR significantly exceeding diagonal values after Bonferroni correction (α_corr_ = 0.0033), while HT-LRT achieves its best performance radially and concentrates its limitation in the sagittal diagonal direction, the only directional contrast surviving correction in HT-LRT (p< 0.0001, d = −1.69). This inversion is geometrically coherent and technique-specific: arc-based VMAT delivery, constrained to a limited number of coplanar arcs, provides less angular modulation in the intra-plane direction, whereas helical delivery achieves superior gradient control precisely in that direction. The limited number of directional pairs surviving Bonferroni correction, restricted to the radial vs diagonal contrast in both techniques, should not be interpreted as a limitation of the directional framework, but rather as an indirect validation of the median VPDR as a summary metric: the majority of directional pairs are dosimetrically homogeneous within each technique, confirming that VPDR_median_ aggregates geometrically equivalent directions and provides a stable and representative estimate of the overall peak-valley modulation pattern. The directional decomposition simultaneously retains the ability to identify the specific geometric axis along which each technique concentrates its dosimetric limitation, information that is inherently invisible to scalar summaries.

The DVH results confirm and extend the VPDR findings at the structural level. The significantly warmer valley doses observed with VMAT-LRT, median GTV D50% 11.03 vs 7.45 Gy, and the concomitantly steeper peak-to-valley gradient with HT-LRT, reflected in higher GTV D1%, are directly concordant with the lower directional VPDR values observed for HT-LRT across all computational methods. The absence of significant differences in GTV prescription isodose coverage excludes the possibility that HT-LRT achieves valley dose suppression at the expense of vertex coverage, a concern that is particularly relevant in the multi-fraction setting where tumor control at the vertex level must be preserved. Normal tissue sparing was superior with HT-LRT at low dose levels, consistent with the superior conformity and reduced low-dose spillage demonstrated for TomoHelical delivery in phantom conditions by Sihawong et al. (22). The approximately 2.5-fold longer beam-on time and 7-fold greater monitor unit demand per fraction observed with HT-LRT represent practical limitations that should be carefully weighed against the dosimetric benefits in individual clinical scenarios, particularly in patients where intrafraction motion represents a significant concern. Previous work has demonstrated that lattice dose distributions are relatively robust to motion amplitudes up to approximately 0.5 cm, with larger displacements leading to progressive degradation of the peak-valley contrast (28), a consideration that gains additional relevance for the longer delivery times inherent to HT-LRT.

The morphological analysis revealed that scalar VPDR metrics are largely insensitive to target geometry in HT-LRT and only weakly sensitive in VMAT-LRT, while directional analysis exposes geometrically motivated and technique-specific associations. The consistent positive correlation between target elongation and radial VPDR in both techniques is physically motivated by an inter-plane dose summation mechanism intrinsic to the staggered triangular lattice geometry: in elongated targets accommodating multiple vertex planes along the major axis, radial valley regions receive cumulative dose contributions from inter-plane vertices positioned above the centroid of each intra-plane triangle, systematically elevating valley doses regardless of delivery modality. The technique-specific sensitivity of radial VPDR to target compactness in HT-LRT, absent in VMAT-LRT, suggests that helical delivery is more responsive to the minor-axis geometry of the target, likely reflecting the more isotropic angular sampling achievable with continuous rotation compared with coplanar arc arrangements. These findings have direct clinical implications for treatment planning: in elongated targets, particular attention should be directed to optimizing radial valley doses, as both techniques are inherently limited in this direction by the geometric dose summation effect; for compact targets treated with HT-LRT, compactness represents an additional morphological predictor of achievable radial VPDR that may guide vertex configuration decisions.

In clinical practice, managing bulky tumors in palliative settings requires balancing therapeutic efficacy with patient life expectancy. Our cohort reflects this reality, showing a significant dose and fractionation reduction in palliative and hemostatic cases. For patients with limited overall survival, protracted radiotherapy schedules impose a heavy burden on QoL due to daily hospital commutes, an area where SFRT offers a key advantage.

Several limitations of the present study should be acknowledged. First, the retrospective paired design, while controlling for patient-specific anatomical variability, does not allow for randomization or prospective clinical endpoint collection. Second, the lattice geometry was deliberately conservative (2 cm GTV contraction, 5 cm Dc-c, 1 cm vertex diameter). It yielded V_vert_/V_GTV_ values of 0.1–0.3%, below the range most frequently reported in the LRT literature (approximately 1–2%, up to 10%) (12, 20). This choice ensured a safety margin between the vertices and the tumor boundary and limited the overlap of dose contributions from neighboring vertices. Because the geometry was identical for both techniques, it does not bias the paired comparison. Since the filling fraction scales with the cube of the vertex diameter, 1.5 cm vertices in the same arrangement would have yielded approximately 0.3–1.0%. However, the sparse lattice resulted in a median of five vertices per case (five or fewer in 13 of 22 cases). This reduced the number of evaluable vertex pairs and the statistical power of the directional analysis, particularly for the X and Y categories, whose findings should be regarded as exploratory. The presented results may also not directly transfer to denser lattice configurations.

Third, inter-vertex valley doses were relatively high, particularly with VMAT-LRT, and in representative cases the 50% isodose bridged adjacent vertices rather than remaining confined around each vertex. This reflects, at least in part, two planning choices. The first was the requirement of near-complete and uniform coverage of each vertex, whereas other experiences normalize the prescription to the median vertex dose, accepting substantially lower doses at the vertex periphery (4, 23); with 1 cm vertices, enforcing full coverage broadens the high-dose region around each vertex and limits the achievable valley depth. The second was the pursuit of a spatially uniform valley: with a 5 cm vertex spacing, homogeneity between distant vertices requires a dose floor, which in this study was bounded by a PTV-Lattice maximum dose objective around 52% of the prescription dose. For the same reason, SBRT-derived criteria developed for isolated targets, such as containment of the 50% isodose within a 2 cm sphere, are not directly transferable to this design.

Fourth, the observed differences may also reflect the planning process. With dedicated VMAT optimization strategies (e.g., tighter valley objectives, vertex-specific ring structures, additional or non-coplanar arcs), lower valley doses and the avoidance of 50–60% isodose bridging are likely achievable, so that part of the HT-LRT advantage may depend on the planning strategy and planner expertise rather than on the delivery technique alone. Moreover, VMAT-LRT and HT-LRT plans were generated with different treatment planning systems and optimization engines, so that differences in optimizer performance cannot be fully separated from those attributable to the delivery technique, and other systems offering dedicated LRT optimization tools may yield different results. All plans were generated by the same planner using standardized templates and were reviewed by two experienced medical physicists, which limits inter-planner variability. Even so, the results should be interpreted as a comparison of the two techniques as implemented with the planning approach and systems described here, rather than as a definitive ranking of delivery platforms.

Furthermore, a relatively homogeneous vertex dose was pursued. This differs from the markedly heterogeneous peak distributions typical of SBRT-like LRT. Allowing higher intra-vertex hotspots would likely have increased peak-to-valley contrast with both techniques, although VPDR_Wu_, which uses the prescription dose as the peak reference, would be unaffected.

Finally, the generalizability of the morphology–VPDR correlations may be limited by the anatomical and volumetric heterogeneity of the cohort. Finally, the study focuses exclusively on dosimetric endpoints and does not address clinical outcomes, biological response, or differential immune-mediated effects of the two delivery techniques. These aspects will require prospective investigation in larger cohorts with systematic clinical follow-up.

## Conclusions

This study provides the first patient-specific dosimetric comparison between HT-LRT and VMAT-LRT, demonstrating, under the planning conditions adopted in this study, a consistent and clinically meaningful advantage of Helical Tomotherapy across scalar VPDR metrics, DVH-based endpoints, and normal tissue sparing, translating into clinical practice the phantom-based findings previously published. Beyond confirming this overall advantage, the directionally-resolved analysis revealed that the dosimetric performance of each technique is not uniform across the three-dimensional lattice geometry, but concentrated along specific spatial axes that reflect the intrinsic delivery characteristics of arc-based and helical rotation, respectively.

This directional heterogeneity, together with the morphology-dependent associations identified for radial VPDR, would remain undetected by scalar summary metrics alone, underscoring the added value of a directionally-resolved analytical framework in LRT dosimetric evaluation. More broadly, these results highlight a methodological issue that extends beyond the specific comparison presented here: the current LRT literature relies on a variety of VPDR definitions, lattice geometries, and reporting conventions that are not yet standardized across institutions. In the absence of harmonized metrics and reporting criteria, comparisons between studies, delivery platforms, and planning strategies risk being incomplete or even misleading, as differences attributed to a given technique may instead reflect underlying methodological heterogeneity. The present work, by applying a uniform planning template and a multi-method, directionally-resolved dosimetric framework to a paired patient cohort, represents a step toward more standardized and reproducible LRT dosimetric assessment, and supports the case for a broader consensus on VPDR definitions and reporting practices to enable meaningful cross-study and cross-platform comparisons. Prospective studies incorporating larger cohorts and clinical outcome data will be needed to translate these dosimetric findings into evidence-based treatment selection criteria for LRT delivery. In our view, the novel SFRT/LRT approach emerges as a particularly compelling therapeutic option for palliative patients with reduced life expectancy. In addition to delivering rapid tumor control and symptom relief, SFRT substantially improves quality of life by compressing total treatment time and minimizing hospital attendance, maximizing both therapeutic index and patient comfort when time is critical.

## Data Availability

The original contributions presented in the study are publicly available. These raw data can be accessed at the Zenodo repository: https://doi.org/10.5281/zenodo.22252066.
