# Supplementary material for: Dosimetric feasibility of lattice radiotherapy for bulky tumors: a paired VMAT vs. helical tomotherapy comparison - the STRYKE trial
Source: Front Oncol. 2026 Oct 27;16:1986573. doi: 10.3389/fonc.2026.1986573 (PMC13747859; doi:10.3389/fonc.2026.1986573)
Supplement: Supplementary file 1 [file Table1.docx]

Supplementary

**Table S1.** Lattice geometry and target volume characteristics across the study cohort. For each case, the center-to-center distance (D_c-c_) between vertex pairs is reported as median (min–max range) in millimeters, stratified by directional category (radial, z, x, y, coronal and sagittal diagonal). The number of vertex pairs evaluated within each directional category is reported in curly brackets {n}. GTV: gross tumor volume; V_vert_/V_GTV_: % VTV to GTV volume ratio.

| **Case** | **N_vert_** | **D_c-c_ by direction — median (range) {n pairs} (mm)** | | | | | | **GTV (cc)** | **V_vert_/V_GTV_ (%)** |
| --- | --- | --- | --- | --- | --- | --- | --- | --- | --- |
|  |  | **Radial** | **Z (SI)** | **X (LR)** | **Y (AP)** | **Diag-coronal** | **Diag-sagittal** |  |  |
| **1** | 12 | 50.4 (49.9-50.8) {7} | 53.8 (53.8-53.8) {2} | 70.8 (70.8-71.2) {3} | 71.0  {1} | 44.7 (44.0-45.1) {9} | 44.5(44.4-45.2)  {7} | 3119.0 | 0.2 |
| **2** | 7 | 51.3 (51.0–51.3) {4} | 54.1  {1} | 72.7 (72.6–72.7) {2} | - | 45.3 (45.1–45.4) {6} | 45.1 (45.0–45.2) {4} | 2021.1 | 0.1 |
| **3** | 21 | 50.4 (49.6–50.7) {13} | 54.0 (53.9–54.1)  {9} | 70.8 (70.7–71.6)  {5} | 70.8  {1} | 44.9 (44.0–45.0)  {21} | 44.6 (44.4–45.0)  {17} | 4927.7 | 0.2 |
| **4** | 4 | 50.4 (50.2–50.5)  {2} | - | - | - | 44.6 (44.4–44.7)  {2} | 44.8 (44.8-44.8)  {2} | 611.9 | 0.3 |
| **5** | 19 | 50.4 (50.0–50.6)  {10} | 54.1 (54.0–54.2)  {4} | 71.6  {1} | 71.1 (71.0–71.2) {2} | 44.8 (44.4–45.0)  {13} | 44.6 (44.0–44.8)  {14} | 4451.6 | 0.2 |
| **6** | 12 | 50.9 (50.7–51.1)  {6} | 54.0 (53.9–54.1)  {7} | - | 71.3  {1} | 45.2 (45.0–45.3)  {9} | 44.7 (44.6–45.0)  {11} | 2082.3 | 0.3 |
| **7** | 4 | 51.1  {1} | 53.9  {1} | - | - | 45.0 (45.0–45.1)  {2} | 45.3 (45.2–45.4)  {2} | 659.4 | 0.3 |
| **8** | 11 | 50.2 (49.8–50.3)  {6} | 54.3 (53.8–54.4)  {5} | 71.3 (71.2–71.4)  {3} | - | 44.9 (44.2–45.1)  {12} | 44.4 (44.3–44.7)  {8} | 2684.1 | 0.1 |
| **^a^9** | 2 | - | - | - | - | - | - | 380.2 | 0.2 |
| **10** | 5 | 49.7 (49.6–49.8)  {2} | 53.8  {1} | - | 70.8  {1} | 44.2 (44.1–44.3)  {2} | 44.5 (44.2–44.6)  {4} | 1404.7 | 0.2 |
| **11** | 4 | 50.4 (50.3–50.5)  {2} | - | 71.2  {1} | - | 44.7 (44.5–44.8)  {2} | 44.6  {1} | 547.5 | 0.3 |
| **12** | 8 | 50.2 (49.9–50.4)  {3} | 54.0 (53.9–54.1)  {2} | 70.6  {1} | - | 44.4 (44.0–44.9)  {7} | 44.9 (44.7–45.1)  {4} | 1717.0 | 0.2 |
| **13** | 3 | 50.3  {1} | - | - | - | 45.1  {1} | 44.3  {1} | 975.2 | 0.1 |
| **14** | 7 | 50.9 (50.8–50.9)  {3} | 54.0 (54.0–54.1)  {3} | - | - | 45.1 (45.0–45.1)  {5} | 44.9 (44.8–44.9)  {5} | 1031.5 | 0.3 |
| **15** | 3 | 50.6  {1} | - | - | - | 45.0  {1} | 44.7  {1} | 620.3 | 0.2 |
| **16** | 3 | - | - | - | - | 44.5  {1} | - | 419.4 | 0.3 |
| **^b^17** | 2 | 40.6  {1} | - | - | - | - | - | 433.5 | 0.2 |
| **^b^18** | 2 | 35.4  {1} | - | - | - | - | - | 394.3 | 0.2 |
| **19** | 2 | 50.6  {1} | - | - | - | - | - | 701.8 | 0.1 |
| **20** | 4 | 49.7  {1} | 53.8  {1} | - | - | 44.1 (44.0–44.2) {2} | 44.4 (44.4-44.4) {2} | 596.5 | 0.3 |
| **21** | 6 | 50.4 (50.3–50.5) {2} | 54.0 (53.8–54.0) {3} | - | - | 44.5 (44.4–44.6) {3} | 44.8 (44.6–45.0) {3} | 776.9 | 0.3 |
| **22** | 5 | 50.3  {1} | 54.0  {1} | - | - | 44.4 (44.3–44.5) {2} | 44.7 (44.3–44.9) {3} | 726.8 | 0.3 |
| **Median (range)** | 5  (2-21) | 50.4  (35.4-51.3) | 54.0  (53.8-54.3) | 71.2  (70.6-72.7) | 71.0  (70.8-71.3) | 44.8  (44.1-45.3) | 44.7  (44.3-45.3) | 751.9  (380.2-4927.7) | 0.2  (0.1-0.3) |

**Abbreviations:** N_vert_, number of lattice vertices; D_c-c_, center-to-center distance between vertex pairs; SI, superior-inferior; LR, left-right; AP, anterior-posterior; Diag coronal, coronal diagonal; Diag sagittal, sagittal diagonal; GTV, gross tumor volume; VTV, total vertex target volume; V_vert_/V_GTV_, percentage VTV to GTV volume ratio.

**Notes:** For each directional category, D_c-c_ is reported as median (range); A dash (-) indicates that no vertex pair was identified within the corresponding directional category for that case, either due to an insufficient number of vertices or to the specific spatial arrangement of the lattice. ^a^Case 9: only two vertices were delineated; no classifiable nearest-neighbor pairs were identified in any directional category. ^b^Cases 17 and 18: radial D_c-c_ values (40.6 mm and 35.4 mm, respectively) deviate from the cohort median, reflecting a patient-specific lattice spacing adapted to smaller target dimensions.

**Table S2.** Comparison of scalar VPDR for VMAT-LRT and HT-LRT stratified by computational method, directional category, and anatomical district. Values are reported as median (range). Three methods were applied: Wu ($D_{mean(95-100)}/D_{\mathrm{pres}}$), D90/D10 on the global lattice DVH and the median pairwise approach (VPDR median). VPDR is decomposed into six directional subcategories (radial, z, x, y, coronal and sagittal diagonal). Statistical comparison by paired t-test or Wilcoxon signed-rank test (Shapiro–Wilk on differences). Effect size expressed as Cohen's d (mean paired difference / SD of differences); values of 0.2, 0.5, and 0.8 indicate small, medium, and large effects respectively; positive values indicate higher VPDR in VMAT-LRT. Significance: *** p<0.001, ** p<0.01, * p<0.05, ns not significant.

| **Parameter** | **n** | **VMAT-LRT** | **HT-LRT** | **p-value** | **Sig.** | **Cohen's d** |
| --- | --- | --- | --- | --- | --- | --- |
|  |  | **Median (min–max)** | |  |  |  |
| **All patients (n = 22)** | | | | | | |
| VPDR Wu | 22 | 0.37 (0.32–0.46) | 0.29 (0.24–0.40) | <0.001 | ******* | 1.59 |
| VPDR D90/D10 | 22 | 0.42 (0.35–0.49) | 0.31 (0.27–0.41) | <0.001 | ******* | 2.07 |
| VPDR median | 21 | 0.54 (0.43–0.68) | 0.46 (0.27–0.56) | <0.001 | ******* | 1.08 |
| *— Radial* | 19 | 0.59 (0.51–0.79) | 0.43 (0.31–0.48) | <0.001 | ******* | 2.84 |
| *— Z (SI)* | 13 | 0.53 (0.45–0.56) | 0.47 (0.40–0.53) | <0.001 | ******* | 1.45 |
| *— X (LR)* | 8 | 0.56 (0.53–0.68) | 0.44 (0.34–0.52) | 0.004 | ****** | 1.52 |
| *— Y (AP)* | 5 | 0.56 (0.52–0.60) | 0.46 (0.44–0.52) | 0.006 | ****** | 2.41 |
| *— Coronal diagonal* | 18 | 0.48 (0.41–0.54) | 0.49 (0.26–0.61) | 0.321 | *ns* | 0.24 |
| *— Sagittal diagonal* | 18 | 0.50 (0.39–0.54) | 0.49 (0.28–0.60) | 0.625 | *ns* | 0.12 |
| **Abdomen (n = 6)** | | | | | | |
| VPDR Wu | 6 | 0.39 (0.36–0.40) | 0.28 (0.24–0.31) | <0.001 | ******* | 3.71 |
| VPDR D90/D10 | 6 | 0.43 (0.39–0.46) | 0.30 (0.27–0.33) | <0.001 | ******* | 4.78 |
| VPDR median | 5 | 0.55 (0.53–0.56) | 0.43 (0.33–0.50) | 0.012 | ***** | 1.94 |
| *— Radial* | 5 | 0.61 (0.55–0.65) | 0.42 (0.33–0.46) | 0.002 | ****** | 3.10 |
| *— Z (SI)* | 4 | 0.55 (0.52–0.56) | 0.42 (0.41–0.53) | 0.027 | ***** | 2.02 |
| *— X (LR)* | *5* | *0.56 (0.55–0.58)* | *0.43 (0.34–0.49)* | *0.004* | **** | *2.76* |
| *— Coronal diagonal* | 5 | 0.50 (0.48–0.52) | 0.41 (0.28–0.49) | 0.034 | ***** | 1.42 |
| *— Sagittal diagonal* | 5 | 0.51 (0.49–0.52) | 0.45 (0.35–0.54) | 0.148 | *ns* | 0.80 |
| **Extremity (n = 9)** | | | | | | |
| VPDR Wu | 9 | 0.38 (0.35–0.46) | 0.30 (0.28–0.40) | 0.002 | ****** | 1.46 |
| VPDR D90/D10 | 9 | 0.42 (0.38–0.49) | 0.31 (0.30–0.41) | <0.001 | ******* | 1.75 |
| VPDR median | 9 | 0.53 (0.48–0.56) | 0.48 (0.45–0.56) | 0.039 | ***** | 1.05 |
| *— Radial* | 9 | 0.59 (0.54–0.79) | 0.44 (0.40–0.48) | <0.001 | ******* | 2.48 |
| *— Z (SI)* | 7 | 0.51 (0.45–0.56) | 0.48 (0.40–0.51) | 0.007 | ****** | 1.51 |
| *— Coronal diagonal* | 9 | 0.48 (0.45–0.54) | 0.50 (0.42–0.61) | 0.652 | *ns* | −0.36 |
| *— Sagittal diagonal* | 9 | 0.51 (0.45–0.54) | 0.52 (0.49–0.60) | 0.066 | *ns* | −0.71 |
| **Thorax (n = 7)** | | | | | | |
| VPDR Wu | 7 | 0.35 (0.32–0.37) | 0.30 (0.27–0.33) | 0.013 | ***** | 1.32 |
| VPDR D90/D10 | 7 | 0.38 (0.35–0.43) | 0.32 (0.28–0.34) | 0.002 | ****** | 1.94 |
| VPDR median | 7 | 0.54 (0.43–0.68) | 0.41 (0.27–0.50) | 0.014 | ***** | 1.31 |
| *— Radial* | 5 | 0.61 (0.51–0.66) | 0.40 (0.31–0.46) | 0.003 | ****** | 2.92 |
| *— Coronal diagonal* | 4 | 0.43 (0.41–0.48) | 0.45 (0.26–0.54) | 0.834 | *ns* | 0.11 |
| *— Sagittal diagonal* | 4 | 0.45 (0.40–0.47) | 0.44 (0.28–0.48) | 0.602 | *ns* | 0.29 |

**Abbreviations:** VPDR, valley-to-peak dose ratio; HT-LRT, helical tomotherapy lattice radiotherapy; VMAT-LRT, volumetric modulated arc therapy lattice radiotherapy; SI, superior–inferior; LR, left–right; AP, anterior–posterior; ns, not significant.

**Notes:** VPDR Wu: D_mean(95–100)_/D_pres_ (Wu et al.). VPDR D_90%_/D_10%_ on the global lattice volume (Borzov et al.). VPDR median: pairwise midpoint approach (Prado et al.), across all vertex pairs. Directional VPDR values are subsets of the pairwise analysis stratified by geometric orientation. n = number of patient pairs with at least one evaluable vertex pair in the corresponding directional category. Y (AP) direction not evaluable in Extremity and Thorax subgroups due to insufficient vertex pairs; Z (SI) direction not evaluable in the Thorax subgroup. Negative Cohen's d values indicate higher VPDR in HT-LRT than VMAT-LRT for that directional category.

Table S3. Within-technique pairwise comparisons of VPDR metrics, reported separately for VMAT-LRT and HT-LRT. Two independent hypothesis families were defined and corrected separately using the Bonferroni method: (i) scalar method comparisons among VPDR_Wu, VPDR_D90/D10, and VPDR_median (k = 3, α_corr = 0.0167); and (ii) directional pairwise VPDR comparisons among the six geometric categories (Radial, Z, X, Coronal diagonal, Sagittal diagonal, Y; k = 15, α_corr = 0.0033). Scalar-vs-directional comparisons were excluded from both correction families as they reflect methodological rather than geometric heterogeneity and are addressed separately. Post-hoc comparisons were performed using the Wilcoxon signed-rank test on all cases with evaluable values in both metrics under comparison. Raw p-values are reported to allow independent assessment; the Sig. column indicates whether each comparison survives the Bonferroni-corrected threshold applicable to its family (sig. = p_raw < α_corr; ns = not significant after correction). Effect size is reported as Cohen's d for paired samples (mean paired difference / SD of differences); positive values indicate higher VPDR for the first-named metric. † Y direction: n = 5 evaluable cases; results are labelled low power and should not be interpreted as evidence for or against directional differences. ‡ X direction: n = 6–8 evaluable cases; no comparison involving X survived Bonferroni correction within the directional family.

| **Pair** | **VMAT-LRT** | | | | **HT-LRT** | | | |
| --- | --- | --- | --- | --- | --- | --- | --- | --- |
|  | **n** | **W** | **p-value** | **Cohen's d** | **n** | **W** | **p-value** | **Cohen's d** |
| **VPDR Scalar methods (k = 3, α_corr = 0.0167)** | | | | | | | | |
| Wu vs D90/D10 | 22 | 0.0 | < 0.0001 | -2.10 | 22 | 1.0 | < 0.0001 | -1.64 |
| Wu vs median | 21 | 0.0 | < 0.0001 | -2.23 | 21 | 1.0 | < 0.0001 | -2.46 |
| D90/D10 vs median | 21 | 0.0 | < 0.0001 | -1.78 | 21 | 1.0 | < 0.0001 | -2.32 |
| **Directional VPDR (k = 15, α_corr = 0.0033)** | | | | | | | | |
| Radial vs Z | 13 | 6.0 | 0.0034 | +1.07 | 13 | 11.0 | 0.0134 | -0.88 |
| Radial vs X | 7 | 1.0 | 0.0312 | +1.14 | 7 | 3.0 | 0.0781 | -1.05 |
| Radial vs Coronal | 17 | 0.0 | < 0.0001 | +1.55 | 17 | 18.0 | 0.0038 | -0.85 |
| Radial vs Sagittal | 17 | 0.0 | < 0.0001 | +1.43 | 17 | 0.0 | < 0.0001 | -1.69 |
| Radial vs Y † | 5 | 0.0 | 0.0625 | +2.42 | 5 | 0.0 | 0.0625 | -1.47 |
| Z vs X ‡ | 6 | 0.0 | 0.0312 | -1.99 | 6 | 5.0 | 0.3125 | -0.45 |
| Z vs Coronal | 13 | 11.0 | 0.0134 | +0.95 | 13 | 32.0 | 0.3757 | -0.25 |
| Z vs Sagittal | 13 | 11.0 | 0.0134 | +0.90 | 13 | 12.0 | 0.0171 | -0.81 |
| Z vs Y † | 5 | 3.0 | 0.3125 | -0.55 | 5 | 5.0 | 0.6250 | -0.33 |
| X vs Coronal ‡ | 7 | 0.0 | 0.0156 | +3.80 | 7 | 0.0 | 0.0156 | +0.94 |
| X vs Sagittal ‡ | 7 | 0.0 | 0.0156 | +2.75 | 7 | 4.0 | 0.1094 | -0.79 |
| X vs Y †‡ | 3 | — | — | — | 3 | — | — | — |
| Coronal vs Sagittal | 18 | 42.0 | 0.0599 | -0.45 | 18 | 41.0 | 0.0539 | -0.49 |
| Coronal vs Y † | 5 | 0.0 | 0.0625 | -1.68 | 5 | 7.0 | 1.0000 | +0.15 |
| Sagittal vs Y † | 5 | 0.0 | 0.0625 | -1.42 | 5 | 6.0 | 0.8125 | +0.15 |

**Abbreviations: VPDR, valley-to-peak dose ratio; VMAT-LRT, volumetric modulated arc therapy lattice radiotherapy; HT-LRT, helical tomotherapy lattice radiotherapy; Z, superior–inferior axis; X, left–right axis; Y, anterior–posterior axis; Coronal, coronal diagonal (inter-plane oblique, z–x plane); Sagittal, sagittal diagonal (inter-plane oblique, z–y plane); W, Wilcoxon statistic; r, rank-biserial correlation coefficient; IQR, interquartile range; p_Bonf, Bonferroni-corrected p-value; *low power*, test performed but statistical power insufficient for interpretation; *not performed*, test not executable due to n<4.**

Table S4. Three-dimensional morphological characterization of gross tumor volumes (n = 22). Values reported as median (min–max). PCA-derived semi-axes and shape indices computed after isotropic resampling.

| Morphological feature | All patients (n=22) | Abdomen (n=6) | Extremity (n=9) | Thorax (n=7) |
| --- | --- | --- | --- | --- |
|  | Median (min–max) | Median (min–max) | Median (min–max) | Median (min–max) |
| **Volume and size** | | | | |
| Volume (cc) | 782 (397–5032) | 2397 (397–5032) | 753 (566–4548) | 451 (417–1442) |
| Surface area (mm²) | 65127 (33575–248704) | 128501 (33575–248704) | 70640 (46465–226810) | 51421 (44660–102378) |
| **PCA semi-axes (mm)** | | | | |
| Semi-axis a (major) | 36.8 (25.0–80.8) | 42.9 (25.0–80.8) | 41.3 (26.9–75.3) | 27.5 (26.3–48.4) |
| Semi-axis b (intermediate) | 26.1 (21.1–50.4) | 40.1 (21.1–50.4) | 26.6 (23.1–50.4) | 24.1 (21.9–33.3) |
| Semi-axis c (minor) | 21.4 (14.9–39.5) | 31.4 (14.9–39.5) | 20.7 (16.5–35.1) | 19.9 (14.9–22.1) |
| **Shape indices (dimensionless)** | | | | |
| Aspect Ratio (a/c) | 1.77 (1.17–3.27) | 1.73 (1.17–2.14) | 1.90 (1.48–3.27) | 1.57 (1.17–2.19) |
| Elongation (b/a) | 0.78 (0.43–0.99) | 0.77 (0.53–0.99) | 0.66 (0.43–0.95) | 0.83 (0.70–0.94) |
| Flatness (c/a) | 0.57 (0.31–0.85) | 0.58 (0.31–0.79) | 0.44 (0.31–0.67) | 0.64 (0.46–0.86) |
| Compactness (c/b) | 0.77 (0.59–0.93) | 0.79 (0.67–0.87) | 0.74 (0.62–0.80) | 0.77 (0.59–0.91) |
| Wadell Sphericity | 0.66 (0.53–0.79) | 0.69 (0.57–0.79) | 0.65 (0.53–0.77) | 0.60 (0.53–0.78) |

**Abbreviations:** PCA, principal component analysis; Aspect Ratio, ratio of major to minor semi-axis (a/c); Elongation, ratio of intermediate to major semi-axis (b/a); Flatness, ratio of minor to major semi-axis (c/a); Compactness, ratio of minor to intermediate semi-axis (c/b); Wadell Sphericity, ratio of the surface area of an equal-volume sphere to the actual surface area. All indices range between 0 and 1, where 1 indicates a perfect sphere. Aspect Ratio ≥ 1, where higher values indicate more elongated geometries.

Table S5. Spearman correlation matrix between morphological features and directional VPDR metrics for VMAT-LRT (upper panel) and HT-LRT (lower panel). Spearman's ρ coefficients are reported for each morphological feature (rows) against each VPDR metric (columns), including three scalar methods (Wu, D90/D10, Median) and six directional pairwise VPDR categories (Radial, Z, X, Coronal diagonal, Sagittal diagonal, Y). Correlations were computed on all cases with evaluable values for both variables in each pair; sample sizes vary by directional category due to patient-specific lattice geometry (Radial n = 19, Z n = 13, X n = 8, Coronal n = 18, Sagittal n = 18, Y n = 5). A positive ρ indicates that higher values of the morphological feature are associated with higher VPDR — i.e., warmer valley doses and less effective spatial fractionation; a negative ρ indicates the opposite. Significance: ** p < 0.01, * p < 0.05; unmarked values are not significant. Morphological features are derived from three-dimensional PCA-based shape analysis after isotropic resampling, as detailed in Table 4. Aspect Ratio = a/c (major to minor semi-axis ratio); Elongation = b/a (intermediate to major); Flatness = c/a (minor to major); Compactness = c/b (minor to intermediate); Wadell Sphericity = ratio of surface area of an equal-volume sphere to actual surface area. Y-direction correlations (n = 5) should be interpreted with caution due to limited statistical power.

| Feature | **VMAT-LRT** | | | | | | | | |
| --- | --- | --- | --- | --- | --- | --- | --- | --- | --- |
|  | VPDR | | | | | | | | |
|  | Wu | D90/D10 | Median | Rad | Z | X | Coro | Sagit | Y |
| Volume (cc) | *0.28* | 0.48^*^ | *0.05* | *0.36* | *0.16* | *-0.10* | 0.55^*^ | *0.27* | -0.60^*^ |
| Semi-axis a (mm) | *0.16* | *0.32* | *00.05* | 0.48^*^ | *-0.22* | *-0.21* | *0.29* | *0.12* | -0.70^*^ |
| Semi-axis b (mm) | *0.18* | 0.43^*^ | *0.09* | *0.18* | *0.26* | *0.00* | 0.49^*^ | *0.21* | -0.60^*^ |
| Semi-axis c (mm) | *0.08* | *0.29* | *0.08* | *0.12* | *0.28* | *0.00* | *0.45* | *0.26* | -0.60^*^ |
| Surface (mm²) | *0.05* | *0.30* | *0.04* | *0.39* | *0.08* | *-0.07* | *0.37* | *0.14* | -0.60^*^ |
| Aspect Ratio (a/c) | *0.00* | *0.03* | *-0.11* | 0.47^*^ | *-0.52* | *-0.48* | *-0.23* | *-0.18* | *0.20* |
| Elongation (b/a) | *0.03* | *0.06* | *0.03* | *-0.44* | *0.45* | *0.36* | *0.09* | *0.03* | *0.40* |
| Flatness (c/a) | *0.00* | *-0.03* | *0.11* | -0.47^*^ | *0.52* | *0.48* | *0.23* | *0.18* | *-0.20* |
| Compactness (c/b) | *0.00* | *-0.10* | *0.07* | *-0.11* | *0.24* | *-0.14* | *0.18* | *0.16* | *-0.10* |
| Wadell Sphericity | 0.51^*^ | 0.49^*^ | *-0.01* | *-0.18* | *0.32* | *-0.07* | 0.48^*^ | *0.37* | 0.90^*^ |

| Feature | **HT-LRT** | | | | | | | | |
| --- | --- | --- | --- | --- | --- | --- | --- | --- | --- |
|  | VPDR | | | | | | | | |
|  | Wu | D90/D10 | Median | Rad | Z | X | Coro | Sagit | Y |
| Volume (cc) | *0.04* | *0.20* | *0.36* | *0.44* | *-0.19* | *0.00* | *0.09* | *0.17* | -0.90^*^ |
| Semi-axis a (mm) | *0.11* | *0.21* | *0.42* | 0.53^*^ | *-0.16* | *0.29* | *0.16* | *0.23* | *-0.80* |
| Semi-axis b (mm) | *0.03* | *0.16* | *0.28* | *0.24* | *-0.23* | *0.14* | *0.03* | *0.04* | -0.90^*^ |
| Semi-axis c (mm) | *-0.15* | *-0.03* | *0.02* | *0.01* | *-0.14* | *0.00* | *-0.20* | *-0.10* | -0.90^*^ |
| Surface (mm²) | *-0.02* | *0.09* | *0.20* | *0.39* | *-0.28* | *0.14* | *-0.06* | *-0.01* | -0.90^*^ |
| Aspect Ratio (a/c) | *0.20* | *0.15* | *0.35* | **0.59^**^** | *-0.01* | *0.50* | *0.26* | *0.27* | *0.30* |
| Elongation (b/a) | *-0.06* | *-0.01* | *-0.23* | *-0.36* | *0.08* | *-0.33* | *-0.19* | *-0.27* | *0.60* |
| Flatness (c/a) | *-0.20* | *-0.15* | *-0.35* | **-0.59^**^** | *0.01* | *-0.50* | *-0.26* | *-0.27* | *-0.30* |
| Compactness (c/b) | *-0.23* | *-0.20* | *-0.32* | -0.46^*^ | *0.05* | *-0.43* | *-0.27* | *-0.10* | *-0.40* |
| Wadell Sphericity | *-0.03* | *0.11* | *0.24* | *0.04* | *0.18* | *-0.33* | *0.12* | *0.21* | *0.60* |
